# PAX7 is a required target for microRNA-206-induced differentiation of fusion-negative rhabdomyosarcoma

**DOI:** 10.1038/cddis.2016.159

**Published:** 2016-06-09

**Authors:** J A Hanna, M R Garcia, J C Go, D Finkelstein, K Kodali, V Pagala, X Wang, J Peng, M E Hatley

**Affiliations:** 1 Department of Oncology, St. Jude Children's Research Hospital, 262 Danny Thomas Place, Memphis, TN 38105, USA; 2 Department of Computational Biology, St. Jude Children's Research Hospital, 262 Danny Thomas Place, Memphis, TN 38105, USA; 3 St. Jude Proteomics Facility, St. Jude Children's Research Hospital, 262 Danny Thomas Place, Memphis, TN 38105, USA; 4 Department of Structural Biology, St. Jude Children's Research Hospital, 262 Danny Thomas Place, Memphis, TN 38105, USA; 5 Department of Developmental Neurobiology, St. Jude Children's Research Hospital, 262 Danny Thomas Place, Memphis, TN 38105, USA

## Abstract

Rhabdomyosarcoma (RMS) is the most common soft tissue sarcoma of childhood. RMS can be parsed based on clinical outcome into two subtypes, fusion-positive RMS (FP-RMS) or fusion-negative RMS (FN-RMS) based on the presence or absence of either *PAX3-FOXO1* or *PAX7-FOXO1* gene fusions. In both RMS subtypes, tumor cells show histology and a gene expression pattern resembling that of developmentally arrested skeletal muscle. Differentiation therapy is an attractive approach to embryonal tumors of childhood including RMS; however, agents to drive RMS differentiation have not entered the clinic and their mechanisms remain unclear. MicroRNA-206 (miR-206) expression increases through normal muscle development and has decreased levels in RMS compared with normal skeletal muscle. Increasing miR-206 expression drives differentiation of RMS, but the target genes responsible for the relief of the development arrest are largely unknown. Using a combinatorial approach with gene and proteomic profiling coupled with genetic rescue, we identified key miR-206 targets responsible for the FN-RMS differentiation blockade, PAX7, PAX3, NOTCH3, and CCND2. Specifically, we determined that PAX7 downregulation is necessary for miR-206-induced cell cycle exit and myogenic differentiation in FN-RMS but not in FP-RMS. Gene knockdown of targets necessary for miR-206-induced differentiation alone or in combination was not sufficient to phenocopy the differentiation phenotype from miR-206, thus illustrating that miR-206 replacement offers the ability to modulate a complex network of genes responsible for the developmental arrest in FN-RMS. Genetic deletion of *miR-206* in a mouse model of FN-RMS accelerated and exacerbated tumor development, indicating that both *in vitro* and *in vivo* miR-206 acts as a tumor suppressor in FN-RMS at least partially through downregulation of PAX7. Collectively, our results illustrate that miR-206 relieves the differentiation arrest in FN-RMS and suggests that miR-206 replacement could be a potential therapeutic differentiation strategy.

Rhabdomyosarcoma (RMS) is the most common soft tissue sarcoma of childhood and is divided into two major histologic subclasses: embryonal RMS (ERMS) and alveolar RMS (ARMS). Most (~80%) ARMS tumors harbor chromosomal translocations resulting in either *PAX3-FOXO1* or *PAX7*-*FOXO1* gene fusions. The presence of the *PAX3/7-FOXO1* fusion gene foretells a worse prognosis and is superior to histology in predicting survival. ARMS patients without a *PAX3/7-FOXO1* translocation have both molecular features and clinical outcome similar to ERMS.^1, 2^ Therefore, molecular classification as fusion-positive RMS (FP-RMS) and fusion-negative RMS (FN-RMS) based on the presence or absence of the *PAX3/7-FOXO1* fusion more accurately represents both the biology and clinical features of RMS. However, despite the recently expanded genomic understanding of RMS, patient survival and the treatment strategies have not changed in decades.^3, 4, 5, 6^

Because of the resemblance to developing skeletal muscle, RMS is often viewed through the prism of normal muscle. Three decades of research have illuminated a tightly controlled process through temporal expression of the myogenic regulatory factors (Mrfs), Myogenic Differentiation 1 (MyoD1), Myf5, Mrf4 (Myf6) and Myogenin to drive skeletal muscle differentiation coupled with a terminal exit from the cell cycle. The transcription factors Pax3 and Pax7 act upstream of the Mrfs in establishing the muscle lineage.^7, 8^ Despite the expression of Mrfs, RMS cells arrest and fail to properly execute terminal muscle differentiation.^9^ FN-RMS cells also maintain high expression of PAX7 and PAX3, transcription factors that promote proliferation and self-renewal in myogenic satellite cells.^10, 11^ However, the full constellation of factors contributing to the differentiation arrest in RMS remains elusive.^12^

MicroRNAs (miRNAs) are non-coding RNAs that reduce gene expression through binding complementary sequences in 3′ untranslated regions (UTR) of target mRNAs resulting in transcript degradation.^13^ miR-206 is a member of a miRNA family with miR-1-1 and miR-1-2 that share an identical seed sequence while differing at four base pairs outside of the seed sequence in the mature miRNA. While miR-1 is expressed more abundantly in cardiac muscle, miR-206 is expressed nearly exclusively in mature skeletal muscle with increasing expression during myogenesis driven by MyoD1 and Myogenin.^14, 15, 16^ Genetic deletion of miR-206 in mice has revealed a role of miR-206 in the regeneration of the neuromuscular synapsis and skeletal muscle regeneration following injury.^17, 18, 19^

In both FN- and FP-RMS, decreased miR-206 expression has been demonstrated in patient tumors compared with normal skeletal muscle.^20, 21^ Higher miR-206 expression correlated to increased patient survival in FN-RMS but not in FP-RMS.^21^ To gain insight into the biological relevance of miR-206 in RMS, several groups overexpressed miR-206 in RMS cell lines and illustrated decreased proliferation and migration as well as an induction of differentiation.^21, 22, 23, 24^ Furthermore, viral expression of miR-206 in RMS cell line xenografts in mice decreased tumor growth.^22, 25^ This recent work has highlighted a few exciting targets of miR-206 in RMS;^26^ however, the necessity and/or sufficiency of these putative miR-206 target genes in mediating RMS differentiation remained unexplored. Using a combinatorial approach with microarrays, large-scale proteomics, and prediction algorithms, we identified the crucial miR-206 targets responsible for the developmental arrest in RMS.

## Results

### miR-206 mimic enforces differentiation of human RMS cell lines

**Figure 1:**
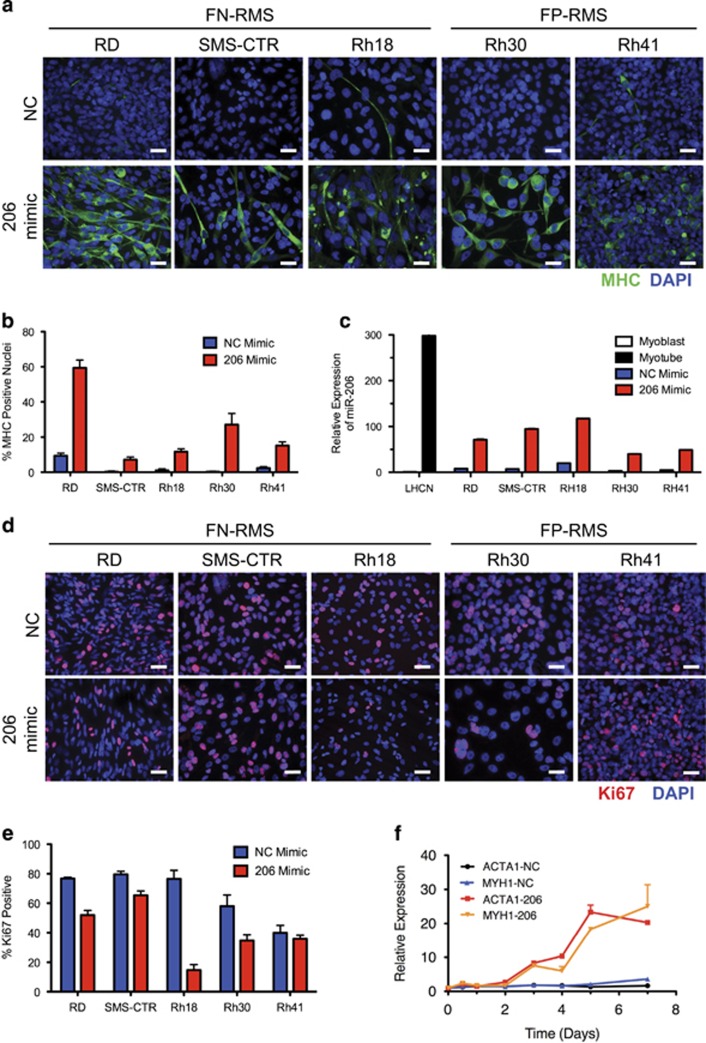
Transfection of miR-206 mimic induces differentiation and decreases proliferation in RMS. (**a**) ICC for MHC (green) and DAPI (blue) in human RMS cell lines 5 days after transfection with NC or miR-206 mimic. (**b**) Quantification of differentiation index or the percentage of MHC-positive nuclei from four representative fields from (**a**). Student's unpaired *t*-test *P*<0.01 for all comparisons between NC and miR-206 mimic. (**c**) miR-206 expression levels by qRT-PCR for cells in (**a**) and LHCN-M2 myoblasts in growth media and LHCN myotubes after 6 days of differentiation, Student's unpaired *t*-test *P*<0.01 for all. (**d**) ICC for Ki67 (red) and DAPI (blue) in human RMS cell lines 5 days after indicated mimic transfection. (**e**) Quantification of proliferation index or the percentage of Ki67-positive nuclei from four representative fields from (**d**). *P*<0.05 for all excluding Rh41 *P*=0.435. (**f**) Time course of *ACTA1* and *MYH1* gene expression by qRT-PCR after mimic transfection at the indicated time points. Scale bars=25 *μ*m. All data represented as mean±S.E.M.

To interrogate the mechanism and spectrum of miR-206 targets important for RMS pathogenesis, a miR-206 mimic or negative control (NC) mimic was transfected into human RMS cell lines. All RMS cell lines have lower miR-206 levels than human myotubes (Supplementary Figure S1a). Transfection of miR-206 mimic induced expression of myosin heavy chain (MHC), a differentiated muscle marker, in all RMS cell lines tested including the FN-RMS cell lines RD, SMS-CTR and Rh18 and the FP-RMS cell lines Rh30 and Rh41 (Figures 1a and b). Cell elongation and fusion was more evident in FN-RMS than in FP-RMS. In addition, miR-206 induced expression of terminal muscle differentiation markers evidenced by increased *MYH1*, *ACTA1* and *CKM* mRNA (Supplementary Figures S1b–d). These changes were accomplished with a physiologic increase in miR-206 levels in cells maintained under high serum growth conditions still below the expression level of miR-206 in differentiated myotubes (Figure 1c and Supplementary Figure S1a). Low serum as used in normal myoblast differentiation experiments was not required for the miR-206-induced RMS differentiation. In addition, increased miR-206 levels led to a decrease in Ki67 expression, a marker of proliferation, in all RMS cell lines but Rh41 (Figures 1d and e). Thus, transfection with a miR-206 mimic relieved the differentiation blockade and decreased cell proliferation in RMS.

Prior to exploring the miR-206 target genes driving the differentiation phenotype, we determined the timing of expression of markers of terminal muscle differentiation following miR-206 transfection. RD cells were transfected with NC or miR-206 mimic and RNA was collected at 12 and 24 h, then daily for 7 days and *ACTA1* and *MYH1* expression was determined by qRT-PCR. As shown in Figure 1f, *ACTA1* and *MYH1* mRNA increased 3 days after transfection. We assumed this increased expression was a consequence of increased miR-206 levels. We chose 48 h and earlier post transfection with miR-206 mimic to identify direct miR-206 targets that relieve the differentiation blockade before the genetic or morphologic features of differentiation were evident.

### Identification of miR-206 target genes

**Figure 2:**
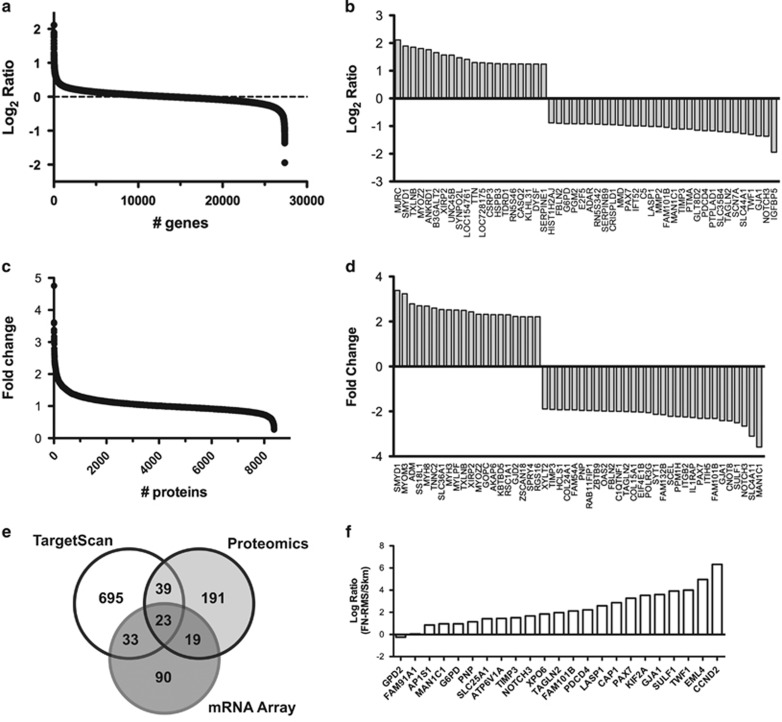
Discovery of miR-206 targets in RMS. RD cells were transfected with miR-206 mimic or NC. RNA and whole-cell lysates were collected at 48 h. (**a**) Gene expression microarrays illustrating the Log_2_ ratio of miR-206 to NC. (**b**) Thirty most downregulated and 20 upregulated genes from (**a**). (**c**) TMT proteomic analysis with fold change. (**d**) Thirty most downregulated and 20 most upregulated proteins from (**c**). (**e**) Venn diagram of mRNAs and proteins decreased by microarray (⩽2-fold) and proteomics (⩽1.3-fold), respectively, upon miR-206 overexpression and miR-206 target genes predicted from TargetScan. (**f**) Gene expression in 77 human FN-RMS tumors compared with 37 normal skeletal muscle samples of the 23 overlapping genes

Next, we sought to identify the miR-206 target genes responsible for relieving the RMS differentiation arrest using a combinatorial approach with RNA microarrays, proteomic profiling and target prediction algorithms. RD cells were transfected with NC or miR-206 mimic and RNA was prepared after 48 h and whole-cell lysates were isolated after 24 and 48 h. Gene expression profiling measured the expression of over 24 000 genes and identified 245 genes with increased expression and 165 genes with decreased levels after transfection with miR-206 with a *P*-value <0.05 and decreased expression with Log_2_ ratio (206/NC)<−0.58 (Figure 2a and Supplementary Figures S2a and b). The top 20 genes with increased expression and the top 30 genes with decreased expression are illustrated in Figure 2b (GEO submitted).

Similarly, out of 8337 total proteins quantified by tandem mass tag (TMT) proteomic profiling, 191 proteins had increased and 272 proteins had decreased levels with a *P*-value of <0.05 and at least a 1.3-fold change after 48 h (Figure 2c). The top 20 proteins with increased levels and top 30 with decreased levels are shown in Figure 2d. Proteomic profiling quantitated 60% (475 of 790) of the predicted miR-206 target genes from TargetScan7.0^27^ with 62 proteins having decreased levels following miR-206 transfection. As well, proteomics quantitated 62% (103 of 165) of genes significantly decreased by microarray (Figure 2e). Of the potential miR-206 targets downregulated by both microarray and proteomics, 23 contained predicted miR-206 sites by TargetScan. Gene ontology analysis of these 23 downregulated genes indicated the most significant genes participate in tissue regeneration and developmental growth (PAX7, NOTCH3 and TIMP3; Supplementary Table S1). In contrast, the same analysis on upregulated genes found no overlapping genes and proteins to have miR-206 predicted binding sites suggesting that the downregulated genes are enriched in miR-206 targets (Supplementary Figure S2c). Ninety-seven proteins had decreased levels 24 h after miR-206 transfection and 32 of these are predicted miR-206 targets (Supplementary Figures S2d–f). Previously documented miR-206 target genes not restricted to RMS, including CCND2, G6PD, GJA1, HDAC4, IGFBP5, NOTCH3 and PAX7, were similarly found to be regulated by either proteomics or microarray.^17, 19, 20, 24, 28, 29^

Decreased miR-206 expression has been shown in RMS compared with normal skeletal muscle.^21^ Therefore, we hypothesized that the most relevant miR-206 targets for RMS pathogenesis and potentially the differentiation arrest of RMS would be upregulated in human RMS tumors (low miR-206) compared with normal muscle (high miR-206). We compared the expression of the group of 23 genes in a cohort of human FN-RMS tumors^1^ to a cohort of human skeletal muscle. All but 2 of the 23 potential target genes had higher expression in FN-RMS tumors compared with skeletal muscle (Figure 2f and Supplementary Figure S2g). A similar pattern was observed comparing FP-RMS tumors with skeletal muscle; however, the ratio for *PAX7* and *NOTCH3* in FP-RMS compared with skeletal muscle was substantially less (Supplementary Figure S2h).

### Validation of putative miR-206 target genes

**Figure 3:**
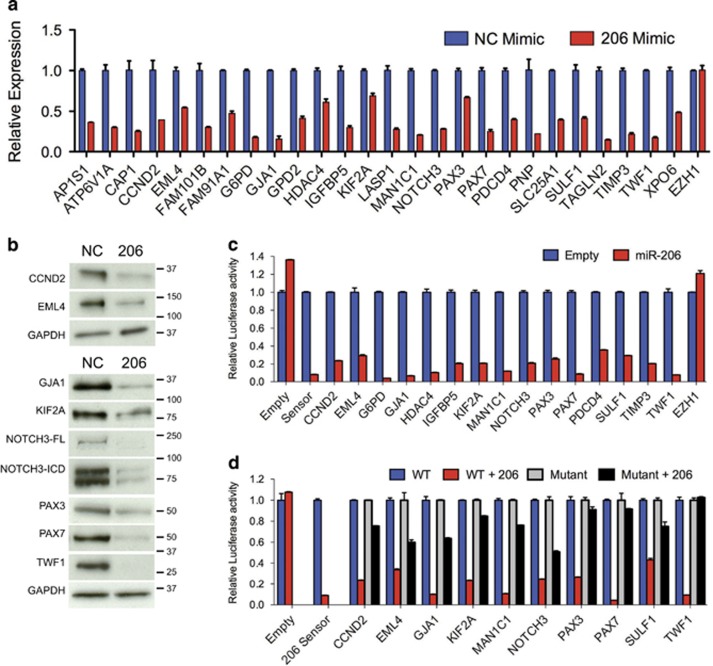
Validation of miR-206 targets. (**a**) qRT-PCR and (**b**) immunoblot analysis of putative miR-206 targets 48 h after NC or miR-206 mimic transfection in RD cells using 5 *μ*g (upper panels) or 25 *μ*g (lower panels) of whole-cell lysate. All differences by qRT-PCR with exception of *EZH1* Student's unpaired *t*-test *P*<0.05. (**c**) Luciferase activity in 293T cells co-transfected with miR-206 or control vector, and empty psiCHECK2 reporter, miR-206 sensor positive control and wild-type 3′ UTRs of indicated genes, including a non-targeting control (EZH1), *P*<0.0001 for all excluding EZH1. (**d**) Luciferase activity in 293T cells co-transfected with miR-206 or control vector and wild type or mutant miR-206 site responsive 3′ UTRs, *P*<0.0005 for all comparing WT and mutant reports with miR-206. Luciferase activity represented as mean±S.E.M. (*n*=4), the Renilla/Firefly luciferase ratio normalized to empty reporter (no miR-206)

The 23 potential miR-206 target genes identified as downregulated by both microarray and proteomics as well as IGFBP5, PAX3 and HDAC4 (previously described miR-206 targets in RMS)^17, 19, 20^ were validated by qRT-PCR (Figure 3a) and a subset by immunoblotting (Figure 3b). All predicted miR-206 targets were significantly downregulated upon transfection with miR-206. In contrast, *EZH1* that does not contain miR-206 recognition sites was unaffected by miR-206 overexpression. Potential targets were validated using 3′ UTR luciferase reporter assays with regulation by miR-206 observed for every target tested compared with the empty psiCHECK2 reporter and the EZH1 3′ UTR negative control (Figure 3c and Supplementary Figure S3a). Specificity of miR-206 regulation was confirmed with dose-dependence as shown for CCND2, NOTCH3, PAX3 and PAX7 (Supplementary Figure S3b) and attenuation when the predicted target sites were mutated (Figure 3d and Supplementary Figure S3c). *CCND2, NOTCH3, PAX3* and *PAX7* expression decreased upon miR-206 mimic transfection in the other FN-RMS (SMS-CTR and Rh18) and FP-RMS (Rh30 and Rh41) cells (Supplementary Figures S3d–g). Using a combinatorial approach with microarray, TMT proteomic profiling and bioinformatic prediction algorithm all predicted miR-206 target genes identified validated; however, the relevance of these miR-206 target genes to the RMS differentiation phenotype had not been determined.

### Genetic rescue identifies miR-206 target genes required for differentiation

To interrogate miR-206 target genes critical for the differentiation arrest in RMS, we attempted to rescue the miR-206-induced phenotype by expressing mutant target genes lacking the 3′ UTR containing miR-206-binding sites with the hypothesis that key targets if unable to be regulated by miR-206 would block miR-206-induced differentiation. We generated lentiviruses to express mutant cDNAs for 9 of the 23 target genes enriched in FN-RMS compared with muscle including the top seven as well as NOTCH3 and G6PD (Figure 2f). In addition, previously described miR-206 targets, IGFBP5, HDAC4 and PAX3, were stably transduced in RD cells. After selection and validation of cDNA overexpression (Supplementary Figure S4a), cells were transfected with NC or miR-206 mimic. While RD cells transduced with the empty vector control retained decreased target gene expression with miR-206 transfection, RD cells overexpressing target gene cDNA lacking the 3′ UTR were not responsive to miR-206 (Supplementary Figures S4a and b).

**Figure 4:**
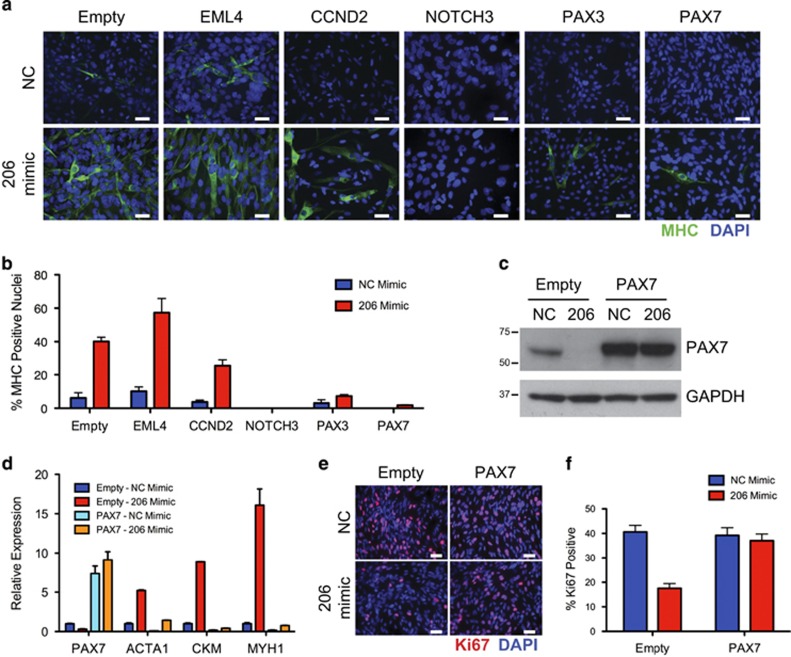
miR-206 regulation of PAX7, PAX3 and NOTCH3 required for differentiation. (**a**) ICC for MHC (green) and DAPI (blue) 5 days after NC or miR-206 mimic transfection in RD cells stably transduced with indicated mutant cDNAs lacking 3′ UTRs. (**b**) Quantification of the differentiation index represented as the percentage of nuclei from (**a**) with MHC-positive cytoplasm, Student's unpaired *t*-test *P*<0.01 for CCND2, NOTCH3, PAX3 and PAX7 cDNAs with miR-206 mimic compared with empty vector. (**c**) PAX7 expression by immunoblot or (**d**) qRT-PCR for *PAX7*, *ACTA1, CKM* and *MYH1* in cells stably transduced with empty vector or mutant PAX7 cDNA as in (**a**). Student's unpaired *t*-test *P*<0.05 for all comparisons of empty vector to PAX7 with miR-206 mimic. (**e**) ICC for Ki67 (red) and DAPI (blue) in NC or miR-206-transfected empty vector or PAX7-transduced cells as in (**a**). (**f**) Quantification of the percentage of cells positive for Ki67. Student's unpaired *t*-test *P*=0.0011 comparing empty vector with PAX7 cells with miR-206 mimic, *P*=0.62 comparing PAX7 cells NC with miR-206 mimic

Muscle differentiation was assessed with MHC immunocytochemistry (ICC) and a full range of phenotypes was observed from enhanced differentiation with EML4, partial inhibition with CCND2, significant block of differentiation with PAX3, and near complete inhibition of differentiation with NOTCH3 and PAX7 (Figures 4a and b and Supplementary Figure S5a). Validation of PAX7 cDNA overexpression and lack of miR-206 regulation was illustrated by qRT-PCR and immunoblotting (Figures 4c and d). Consistent with the MHC ICC, expression of *PAX7*, *PAX3* and *NOTCH3* mutant cDNAs alone blocked the miR-206-induced expression of *CKM* by qRT-PCR (Figure 4d and Supplementary Figure S5d). Although CCND2 partially blocked miR-206-induced differentiation by ICC, CCND2 did not block miR-206-induced *CKM* expression. G6PD (previously shown to delay differentiation in RD18 cells, a subclone of RD)^24^ did have a subtle but significant decrease in *CKM* expression but no detectable difference in MHC expression by ICC (Supplementary Figures S5a–d). These results suggest that downregulation of PAX7, PAX3, CCND2 and NOTCH3 is necessary for the miR-206-induced differentiation of FN-RMS cells.

During normal myogenesis, induction of the differentiation program is coupled with a terminal cell cycle exit. We found partial rescue of Ki67 levels in miR-206 mimic-transfected cells with *CCND2*, *G6PD*, *KIF2A* and *PAX3* mutant cDNA overexpression (Supplementary Figures S5a and c). Overexpression of the mutant *PAX7* cDNA was the only miR-206 target that completely blocked miR-206-induced decreased proliferation (Figures 4e and f and Supplementary Figures S5a and c). NOTCH3-ICD overexpression alone decreased proliferation of RD cells compared with empty control. Paradoxically, miR-206 mimic in NOTCH3-ICD-overexpressing cells increased proliferation. Given the proliferative phenotype with NOTCH3-ICD expression in the absence of miR-206, we focused on PAX7. In order to ensure that the observed phenotypes with mutant PAX7 were not just a result of supraphysiologic protein overexpression, the viral titer was decreased to reduce the protein level of exogenous mutant PAX7 (Supplementary Figure S6a). Stably transduced cells with the lowest titer (a 40-fold reduction) were then transfected with NC or miR-206 mimic. Similar to the original observation even with a subtle increase in PAX7 expression, differentiation was significantly reduced and proliferation was unchanged (Supplementary Figures S6b–g).

**Figure 5:**
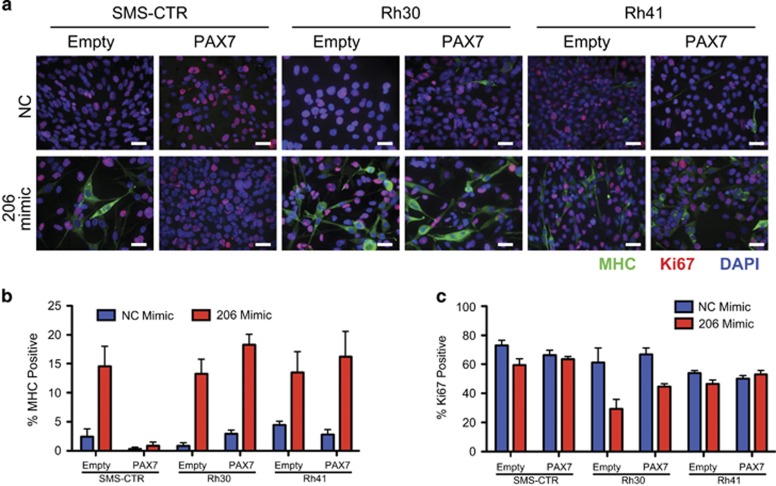
PAX7 knockdown is necessary for miR-206-induced differentiation of FN-RMS but not of FP-RMS. (**a**) ICC for MHC (green), Ki67 (red) and DAPI (blue) 5 days after transfection with NC or miR-206 mimic in SMS-CTR, Rh30 and Rh41 cells transduced with empty vector or PAX7 cDNA lacking 3′ UTR. (**b**) Quantification of the percentage of MHC-positive nuclei from (**a**), in comparing empty vector with PAX7 cells with miR-206 mimic SMS-CTR (*P*=0.0088), Rh30 (*P*=0.15) and Rh41 (*P*=0.64). (**c**) Quantification of Ki67-positive nuclei from (**a**), in comparing empty vector to PAX7 cells with miR-206 mimic SMS-CTR (*P*=0.02), Rh30 (*P*=0.06) and Rh41 (*P*=0.14)

PAX7 expression decreases during normal myogenic differentiation and is highly expressed in FN-RMS but not in FP-RMS similar to previous reports (Supplementary Figure S7a).^30, 31^ To determine the broad role of PAX7 regulation by miR-206 in RMS, mutant PAX7 was overexpressed in a second FN-RMS cell line, SMS-CTR, and two FP-RMS lines, Rh30 and Rh41 (Supplementary Figure S7b). PAX7 mutant overexpression blocked miR-206-induced differentiation measured by MHC ICC and *CKM* expression as well as decreased proliferation maintaining a high Ki67 index in the SMS-CTR cells (Figures 5a–c and Supplementary Figure S7c). However, PAX7 mutant overexpression did not block miR-206-induced differentiation in the FP-RMS cell lines, suggesting PAX7 expression to be a key component to the differentiation block of FN-RMS, but not of FP-RMS.

### Insufficiency of miR-206 targets for differentiation induction

**Figure 6:**
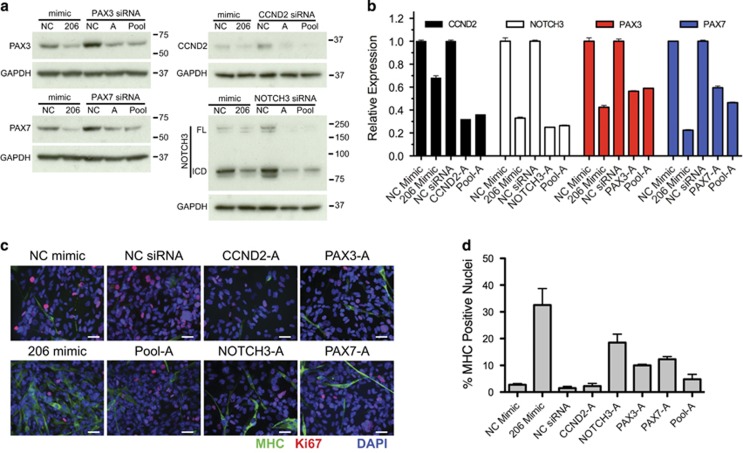
Knockdown of selected miR-206 targets is not sufficient to phenocopy miR-206-induced differentiation. (**a**) Immunoblot and (**b**) qRT-PCR validation of knockdown in RD cells 5 days following transfection with NC mimic, miR-206 mimic, NC siRNA, single A siRNAs, or pool-A of siRNAs to CCND2, NOTCH3, PAX3 and PAX7. Student's unpaired *t*-test *P*<0.01 for all comparisons of miR-206 mimic to NC and target siRNA or pooled siRNAs to NC siRNA by qRT-PCR. (**c**) ICC for MHC (green), Ki67 (red) and DAPI (blue) in transfected cells as in (**a**). (**d**) Quantitation of ICC for the percentage of MHC-positive nuclei, *P*<0.01 for miR-206 mimic, NOTCH3-A, PAX3-A, and PAX7-A siRNAs and *P*=0.56 for CCND2 and *P*=0.17 for Pool-A compared with NC

Because PAX7, PAX3, CCND2 and NOTCH3 downregulation was necessary for miR-206-induced differentiation, we sought to determine if knocking down these genes alone or in combination with siRNAs could phenocopy the miR-206-induced differentiation of RMS. Three siRNAs for each gene were assessed and significant knockdown by qRT-PCR and immunoblotting was observed (Supplementary Figures S8a and b). The best two siRNAs, designated A and B for each gene, were then assessed for their effect on RD cell differentiation (Figures 6a–d). Modest increases in MHC expression were observed with the best siRNAs alone and in a pooled combination of the best siRNA for each of the four genes but not to the same extent as miR-206 mimic (Figures 6c and d). Despite strong knockdown comparable to the downregulation observed with miR-206 mimic, only siRNA knockdown of NOTCH3, PAX3 and PAX7 resulted in a significantly increased MHC staining. Another independent group of siRNAs individually and in a pooled combination resulted in slight increases in MHC and differentiation index with only knockdown of PAX3 and PAX7 alone reaching statistical significance (Supplementary Figures S8c and d). Thus, targeted knockdown of miR-206 targets CCND2, PAX3, PAX7 and NOTCH3 with siRNAs alone or in combination was insufficient to phenocopy miR-206-induced differentiation. However, knockdown of these genes was sufficient to decrease proliferation as measured by Ki67 ICC (Figure 6c and Supplementary Figures S8c and e), suggesting these targets may be contributing to maintaining a high proliferation rate of FN-RMS.

### miR-206 participates in FN-RMS pathogenesis *in vivo*

**Figure 7:**
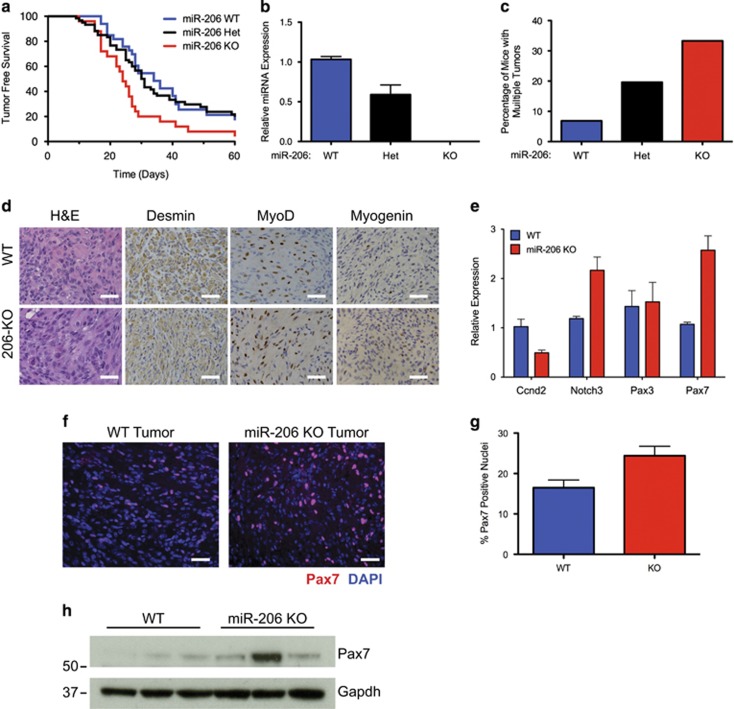
Deletion of miR-206 in an aP2-Cre;SmoM2 FN-RMS model accelerates tumor formation. (**a**) Kaplan–Meier survival curves illustrating tumor-free survival of *aP2-Cre;Smo*^*M2/+*^*;miR-206*^*−/−*^ (red, *n*=25), *aP2-Cre;Smo*^*M2/+*^*;miR-206*^*+/−*^ (black, *n*=60) and *aP2-Cre;Smo*^*M2/+*^*;miR-206*^+/+^ (blue, *n*=33). Median survival WT=34 days, Het=30.5 days, KO=24 days (log rank test *P*=0.027). (**b**) miR-206 expression by qRT-PCR in tumors from each genotype (*n*=3). (**c**) Deletion of miR-206 increases the percentage of mice with multiple tumors. (**d**) Histology of miR-206 WT and KO tumors with H&E and immunostaining with Desmin, MyoD1 and Myogenin. Scale bar is 25 *μ*m. (**e**) Expression of miR-206 targets by qRT-PCR in WT and miR-206 KO tumors (*n*=4), Student's unpaired *t*-test *P*<0.005 for Notch3 and Pax7. (**f**) Immunofluorescent staining of Pax7 (red) and DAPI (blue) in a representative miR-206 WT and KO tumor. Scale bar is 25 *μ*m. (**g**) Quantification for the percentage of positive Pax7 nuclei in (**f**), *P*=0.0135. (**h**) Expression of Pax7 by immunoblot in WT and miR-206 KO tumors using the Sigma AV32742 antibody

To determine if miR-206 contributes to the pathogenesis of RMS *in vivo*, we utilized miR-206 loss of function mice^17^ in a previously described mouse model of FN-RMS.^32^ We bred *miR-206*^*+/−*^; *Smo*^*M2/M2*^ to *aP2-Cre*; *miR-206*^*+/−*^ mice to compare tumor onset kinetics in littermate *miR-206*^+/+^ wild type (WT), *miR-206*^*+/−*^ heterozygous (Het) and *miR-206*^*−/−*^ homozygous null (KO) mice that are also *aP2-Cre; Smo*^*M2/+*^ (Supplementary Figure S9a). miR-206 KO mice had a significant decrease in latency to tumor onset with a median onset of 24 days compared with 34 days in miR-206 WT animals (Figure 7a). In addition, the penetrance of tumors increased to 92% in miR-206 KO mice compared with 85% in WT animals. As expected, miR-206 KO animals expressed no detectable miR-206 and heterozygous miR-206 mice expressed an intermediate level of miR-206 (Figure 7b). The frequency of multiple primary tumors per animal increased with miR-206 loss, suggesting miR-206 loss lowered the threshold for tumor formation (Figure 7c). The histology and IHC staining pattern as well as the expression of myogenic genes in miR-206 KO tumors were indistinguishable from the miR-206 WT tumors in regard to these myogenic features (Figure 7d and Supplementary Figures S9b–d). Interestingly, miR-206 target genes *Notch3* and *Pax7* were significantly upregulated in miR-206 KO tumors compared with WT tumors, thus confirming the *in vitro* target gene regulation identified in human RD cells (Figure 7e). Furthermore, Pax7 protein levels were increased in miR-206 KO tumors by immunofluorescent staining and immunoblot (Figures 7f–h). These results indicate that miR-206 loss accelerated FN-RMS tumor development, and miR-206 actively participates in the biology of FN-RMS.

## Discussion

**Figure 8:**
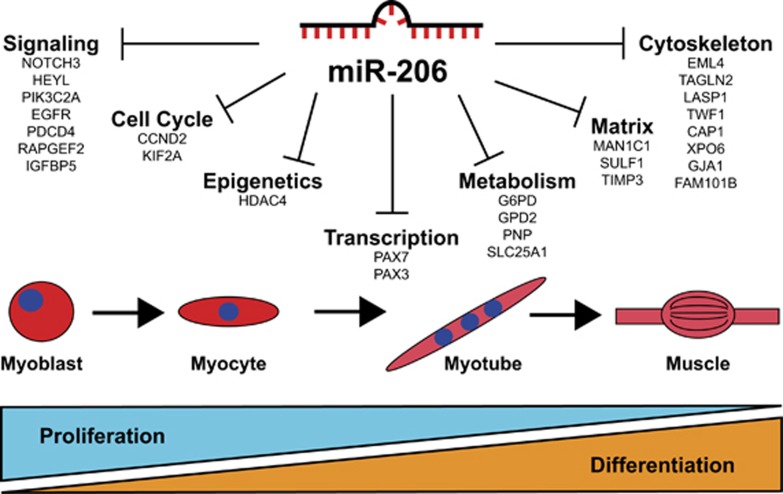
Role of miR-206 and target genes in RMS and muscle differentiation. Targets of miR-206 validate in this report and by others have diverse roles in multiple cellular processes involved in skeletal muscle differentiation

In this study, we demonstrate that miR-206 overexpression with a miRNA mimic led to induction of muscle differentiation coupled with decreased proliferation in FP-RMS and FN-RMS cell lines. An orthogonal approach using a combination of gene arrays, TMT proteomic profiling and miRNA target prediction algorithms identified a subset of 23 genes regulated by miR-206 at the RNA and protein levels. We also established the *in vivo* role of miR-206 in a genetically engineered mouse model of FN-RMS where miR-206 deletion led to an increase in penetrance, number of primary tumors and decreased tumor-free survival. The robust phenotype of miR-206-induced differentiation offers an opportunity to comprehensively explore necessary miR-206 target genes, and thus define the roadblocks to myogenic differentiation in RMS. This study and that of others provide evidence for a model where miR-206 promotes cell cycle exit and differentiation through a collection of targets, including cell cycle regulators, myogenic transcription factors, cytoskeleton proteins as well as proteins involved in metabolism, signaling and epigenetic modification (Figure 8).

Our studies directed focus to CCND2, NOTCH3, PAX3 and PAX7 as key miR-206 targets in FN-RMS. CCND2 is the predominant D-type cyclin in FN-RMS.^33^ Furthermore, miR-206 downregulates CCND2 in both FN- and FP-RMS potentially promoting cell cycle exit.^20^ NOTCH signaling is also increased in RMS and NOTCH inhibition promotes RMS differentiation.^34, 35, 36^ The paired-box transcription factors, PAX3 and PAX7, are expressed in embryonic muscle progenitors and function upstream of Myf5 and MyoD1 to promote myoblast and satellite cell proliferation and self-renewal.^7^ PAX3/PAX7 are normally downregulated in myogenic differentiation and their exogenous overexpression inhibits differentiation.^18, 37, 38^ In addition, mouse satellite cells marked with higher expression of Pax7-nGFP are less likely to undergo myogenic commitment.^39^ PAX3 and PAX7 are overexpressed in FN-RMS and are the targets of reciprocal chromosome translocation with FOXO1 that drives FP-RMS. In addition, PAX3-3′ UTR variants have been identified in RMS and different satellite cell populations, implicating the importance of regulation by miR-206.^20, 40^ Recent investigations of PAX7 in RMS have revealed that PAX7 promotes proliferation through downregulation of MyoD1 in a proteasome-dependent manner.^11^ PAX7 promotes metastasis and invasiveness of RMS and pleomorphic sarcomas.^10, 41^ Surprisingly, the precise role of miR-206 regulation of PAX7 in FN-RMS has remained largely unexplored.

PAX7 was able to significantly inhibit both differentiation and decreased proliferation in response to miR-206 overexpression highlighting the dual roles of miR-206. While induction of differentiation is obviously critical for normal myogenesis, decreased proliferation and cell cycle exit are also important and likely contribute to the tumor-suppressing potential of miR-206. This study highlights the role of PAX7 in RMS. While expression of mutant PAX7 without miR-206-binding sites was able to block both miR-206-induced differentiation and reduced proliferation in FN-RMS, PAX7 did not rescue miR-206 differentiation in FP-RMS. These results emphasize the diversity of genetic drivers between FN-RMS and FP-RMS. FP-RMS tumors are driven by a fusion protein from translocations of either *PAX3* or *PAX7* with *FOXO1*. The *PAX3/7-FOXO1* fusion gene contains the 3′ region of *FOXO1*, including the transactivation domain and 3′ UTR.^9^ Therefore, both the *PAX3-* and *PAX7-FOXO1* lack the 3′ UTR of the *PAX* gene containing miR-206 recognition sites. This results in PAX3/7 target gene expression driven by the fusion protein uncoupled from miR-206 regulation. Despite the distinct difference in drivers of tumorigenesis, miR-206 is able to promote differentiation and cell cycle exit in both subtypes of RMS illustrating that miR-206 RMS phenotypes are secondary to more than PAX7 regulation alone.

To determine whether miR-206-induced differentiation could be explained by a single target gene, we assessed siRNA knockdown of targets. Gene knockdown of target gene alone or in combination only slightly induced differentiation compared with miR-206 treatment, despite knockdown levels comparable to their miR-206-directed downregulation. Knockdown of these targets reduced proliferation as previously shown.^11, 20, 24, 34, 35^ This finding suggests that single targets and even a subset of targets are insufficient to induce differentiation to the extent induced by miR-206 overexpression. Thus, target identification collectively is a key to understanding the mechanism of miR-206-induced differentiation in RMS further suggesting the ‘1 miRNA-1 target' model to be insufficient to explain complex phenotypes induced by miRNAs.^42^

Our results further substantiate the hypothesis that because a single miRNA can target hundreds of mRNAs, they may be more valuable therapeutically than directed agents against a single molecule or pathway. With the recent entry of miRNA mimics into clinical use (NCT01829971) as well as the use of miRNAs as prognostic and diagnostic biomarkers, elucidating the collective targets of a therapeutic miRNA is critical for understanding mechanism and identifying targets for efficacy analysis. The current barrier for broad clinical use of miRNA mimics and inhibitors is delivery, yet as these issues are overcome with new delivery strategies the use of therapeutic miRNAs will advance future targeted therapies in cancer.

miR-206 is potentially therapeutically valuable especially in RMS where it can both promote cell cycle exit and differentiation. miR-206 replacement therapy with a miRNA mimic would allow for regulation of these processes by targeting many nodes in the RMS developmental arrest as well as a mechanism to target transcription factors that have been historically difficult to target with small molecules. In addition, miR-206 has been shown to have tumor suppression capabilities in other cancer types such as breast cancer, lung cancer, hepatocellular carcinoma, pancreatic adenocarcinoma and others.^26^ These studies provide further evidence for the role of miR-206 in decreasing proliferation independent of myogenic differentiation, suggesting miR-206 mimics may be of value as a broad cancer therapy.

## Materials and Methods

### Cell lines

Cell lines were from the following sources: RD (CCL-136) and Rh30 (CRL-2061) cells from ATCC (Manassas, VA, USA), HSMM (CC-2580; Lonza, Basel, Switzerland), LHCN-M2 (Woodring Wright, University of Texas Southwestern Medical Center, Dallas, TX, USA), Rh18 (Children's Oncology Group Cell Line Repository, Monrovia, CA, USA), SMS-CTR (Rene Galindo, University of Texas Southwestern Medical Center), Rh41 (Gerard Grosveld, St Jude) and 293 T (Martine F Roussel, St Jude). RMS lines were authenticated with short tandem repeat profiling (Supplementary Table S2).^43^ Cells were maintained in DMEM (SH30243; HyClone, Logan, UT, USA) supplemented with 10% FBS (HyClone), antibiotic–antimycotic (A5955; Sigma-Aldrich, St Louis, MO, USA) and incubated at 37 °C, 5% CO_2_.

### Mouse strains

All mice used for this study were maintained in mixed genetic backgrounds; thus, littermate controls were used for all comparisons. All strains were described previously: aP2-Cre,^44^ SmoM2 (#5130, Jackson Laboratories, Bar Harbor, ME, USA),^45^ miR-206^−/−^.^17^ All experiments involving animal procedures were reviewed and approved by the SJCRH Institutional Animal Care and Use Committee.

### MicroRNA mimic and siRNA transfections

Cells were transfected with miRVana miRNA Negative Control (4464058; Ambion, Carlsbad, CA, USA) or miR-206 miRVana miRNA mimic (MC10409; Ambion/Thermo Fisher Scientific, Waltham, MA, USA) and Silencer Select siRNAs (Ambion) following the manufacturer's instruction with Lipofectamine RNAiMax (13778075; Invitrogen/Thermo Fisher Scientific, Waltham, MA, USA). siRNAs for CCND2 (s2518-A, s2519-B and s2520-C), NOTCH3 (s9640-A, s9641-B s9642-C), PAX3 (s10058-A, s10059-B s10060-C) and PAX7 (s10070-C, s10071-B and s10072-A) were used. Cells were transfected with 30 nm of mimics or siRNAs.

### Immunocytochemistry and immunohistochemistry

ICC was performed on cells grown on coverslips 5 days after transfection. Cells were fixed in 4% paraformaldehyde, permeabilized in 0.1% Triton X-100 and blocked in 15% normal goat serum (10000C; Invitrogen). Primary and secondary antibodies (Supplementary Table S3) were serially incubated for 1 h at room temperature. Coverslips were mounted with VectaShield with DAPI (H-1500; Vector Laboratories, Burlingame, CA, USA). Images captured with a Nikon Eclipse 80i (Nikon, Tokyo, Japan) at a magnification of × 40. Immunohistochemistry performed as previously described using antibodies for Desmin (Thermo Fisher Scientific), MyoD1 (Cell Marque, Rocklin, CA, USA) and Myogenin (Dako, Carpinteria, CA, USA).^32^ For Pax7 IHC in tumors, PAX7 was detected using the Mouse on Mouse (M.O.M.) kit following the manufacturer's instructions (BMK-2202; Vector Laboratories); however, the signal was detected with the TSA-Plus Cyanine 5 System (NEL745001KT; Perkin Elmer, Waltham, MA, USA) following the manufacturer's instructions.

### RNA isolation and gene expression

Total RNA was isolated using the miRNEasy mini kit (217004; Qiagen, Valencia, CA, USA) according to the manufacturer's instruction. Reverse transcription and quantitative qPCR was performed as previously described.^46^ SYBR primer sequences and Taqman probes are detailed (Supplementary Table S4). The GeneChip Human Gene 2.0 ST Microarray (902499; Affymetrix, Santa Clara, CA, USA) was utilized for gene expression analysis in RD cells 48 h after NC or miR-206 mimic transfection (*n*=4). Normalized signal data were log_2_ transformed in STATA/SE 11.2 (StataCorp, College Station, TX, USA). The log_2_ transformed NC and miR-206 data were batch corrected and compared. Each probeset was compared by an unequal variance *t*-test (Partek Inc., Partek Genomics Suite 6.6, St Louis, MO, USA). Probesets with adjusted *P*-value less than 0.05, an FDR<0.05 and a fold change>2 were *Z-*score normalized, hierarchically clustered and visualized in Spotfire Decision Site 9.2 (TIBCO, Boston, MA, USA). Previously described cohorts of human RMS^1^ and normal skeletal muscle (GEO, GSE9103) were compared by taking the RMS/wt-skeletal muscle log ratios median normalized to center at zero.

### TMT proteomics profiling by liquid chromatography and mass spectrometry (LC-MS/MS)

Cell pellets from 24 and 48 h after NC or miR-206 mimic transfection were lysed, digested and labeled as described previously.^47^ One hundred micrograms of each sample was digested with Lys-C (Wako, Richmond, VA, USA; 1:100 w/w) at room temperature for 2 h, diluted 4 × with 50 mM HEPES and further digested with trypsin (1:50 w/w; Promega, Madison, WI, USA) overnight at room temperature. Peptide samples were acidified by TFA, desalted with Sep-Pak C_18_ cartridge (Waters, Milford, MA, USA), eluted by 60% ACN 1% formic acid and dried by speedvac. Samples were resuspended in 50 mM HEPES and labeled with 10-plex TMT reagents (Thermo Fisher Scientific). The labeled samples were equally pooled, desalted and dried by speedvac. Labeled peptides were analyzed based on an optimized LC-MS/MS platform on an Orbitrap Elite mass spectrometer (Thermo Fisher Scientific).^48^ Acquired MS data were searched against a human protein database utilizing JUMP algorithm, a tag-based database search program, for protein identification (protein FDR<1%).^48^ Quantification was achieved by analyzing the reporter ion intensities from the MS/MS spectra.

### Immunoblotting

Immunoblotting was performed on whole-cell extracts as described previously.^46^ Antibodies applied overnight as detailed in Supplementary Table S3. HRP-conjugated secondary goat anti-mouse (170-1616; Bio-Rad, Hercules, CA, USA) and goat anti-rabbit (170-1615; Bio-Rad) were then visualized with luminol reagent (SC-2048; Santa Cruz Biotechnology, Dallas, TX, USA).

### Molecular cloning of 3′ UTRs and luciferase reporter assays

The 167 base pair genomic fragment of miR-206 was PCR amplified from HSMM genomic DNA using the following primers modified from Taulli *et al.*,^22^ and sublconed into the pCMV6 expression plasmid using the *Eco*RI and SalI sites to generate pCMV6-miR-206. The miR-206 sensor was constructed by inserting a perfect reverse complement of mature miR-206 in psiCHECK2 (C8021; Promega) using the following annealed oligos (Integrated DNA Technologies (IDT), Coralville, IA, USA), Fwd Oligo: 5′-TCG AGC CAC ACA CTT CCT TAC ATT CCA GC-3′ and Rev oligo: 5′-GGC CGC TGG AAT GTA AGG AAGTGT GTG GC-3′ cloned into the *Xho*I/NotI sites of psiCHECK2 to generate the psiCHECK2-miR-206 sensor. The G6PD 3′UTR was subcloned from the pAG68-G6PD-3′UTR vector, a gift from David Bartel (Addgene; plasmid #12020) by digestion with *Sac*I (New England Biolabs (NEB), Ipswich, MA, USA), blunted end with Klenow DNA polymerase (NEB), then digesting with NotI (NEB). The resulting fragment was then ligated to psiCHECK2 that had been digested with *Xho*I (NEB), blunted with Klenow DNA polymerase and then digested with NotI*.* For mutating miR-206 recognition sites in *PAX7* and *CCND2,* gene blocks (IDT) were purchased with all 5 and 3 miR-206 sites mutated, respectively. The 3′ UTR of putative target genes were amplified from HSMM genomic DNA by PCR with and subcloned into psiCHECK2 (C8021; Promega) (Supplementary Table S5). miR-206 recognition sites were mutated with the Quick Change II Site Directed Mutagenesis (200524; Agilent Technologies, Santa Clara, CA, USA) (Supplementary Tables S6 and S7). Luciferase assays were performed by co-transfecting 293 T cells with 100 ng pCMV6-empty or pCMV6-miR-206 with 10 ng of psiCHECK2 reporter plasmids using FuGENE6 (E2691; Promega) and performing the Dual-Luciferase Reporter Assay System (E1910; Promega) after 48 h.

### Molecular cloning of cDNA expression vectors and lentiviral transduction

cDNA expression plasmids were purchased from Addgene or GE Dharmacon Open Biosystems (Lafayette, CO, USA), amplified with PCR and subcloned into pSIN-EF2-Blast as shown in Supplementary Table S8. HDAC4 cDNA was blunt-end cloned from pCMV-SPORT-6 (Open Biosystems) into pSIN-EF2-Blast digested with *Xma*I (NEB). cDNAs for CCND2, EML4, HDAC4, PAX7, PAX3 and TWF1 were cloned from plasmids purchased from GE Dharmacon Open Biosystems. Addgene plasmids G6PD/pRK5 (gift from Xiaolu Yang, #41521),^49^ plenti6.3/hCx43-stop (gift from Robin Shaw, #27383),^50^ pcDNA3-IGFBP5-V5 (gift from Steven Johnson, #11608), pEGFP-KIF2A (gift from Gohta Goshima and Ryota Uehara, #52401),^51^ hNICD3(3xFLAG)-pCDF1-MCS2-EF1-copGFP (gift from Brenda Lilly, #40640),^52^ and pcDNA3.1/Myc-His(-)-HSulf1 (gift from Steven Rosen, #13003)^53^ were used. For NOTCH3, the intracellular domain (NICD) was overexpressed as described previously.^54^

Lentiviruses were generated using the modified pSIN-EF2 lentiviral vector (Addgene; #16578) with blasticidin resistance gene and packaged in 293 T cells as previously described. Virus containing supernatant was used to transduce RMS cells that were then maintained in 5 *μ*g/ml blasticidin.

## Abbreviations

RMS: rhabdomyosarcoma
ERMS: embryonal rhabdomyosarcoma
PAX3: paired-box 3
FOXO1: forkhead box 01
PAX7: paired-box 7
ARMS: alveolar rhabdomyosarcoma
FP-RMS: fusion-positive rhabdomyosarcoma
FN-RMS: fusion-negative rhabdomyosarcoma
Mrfs: myogenic regulatory factors
MyoD1: myogenic differentiation 1
Myf5: myogenic factor 5
Myf6: myogenic factor 6
miRNAs: microRNAs
UTR: untranslated region
NC: negative control
MHC: myosin heavy chain
MYH1: myosin, heavy chain 1, skeletal muscle, adult
ACTA1: actin, alpha 1, skeletal muscle
CKM: creatine kinase, muscle
Ki67: marker of proliferation Ki67
qRT-PCR: quantitative real-time polymerase chain reaction
TMT: tandem mass tag
RNA: ribonucleic acid
TIMP3: tissue inhibitor of metalloproteinases 3
CCND2: cyclin D2
G6PD: glucose-6-phosphate dehydrogenase
GJA1: gap junction protein, alpha 1
HDAC4: histone deacetylase 4
IGFBP5: insulin like growth factor binding protein 5
EZH1: enhancer of zeste 1 polycomb repressive complex 2 subunit
ICC: immunocytochemistry
EML4: echinoderm microtubule associated protein like 4
KIF2a: kinesin heavy chain member 2A
NOTCH3-ICD: NOTCH3 intracellular domain
siRNA: small interfering RNA
KO: knockout
WT: wild type
HET: heterozygous
IHC: immunohistochemistry
DAPI: 4',6-diamidino-2-phenylindole
GAPDH: glyceraldehyde-3-phosphate dehydrogenase

## Supplementary Material

Supplementary Informations
