# Supplementary material for: PAX7 is a required target for microRNA-206-induced differentiation of fusion-negative rhabdomyosarcoma
Source: Cell Death Dis. 2016 Jun 9;7(6):e2256–. doi: 10.1038/cddis.2016.159 (PMC5143388; doi:10.1038/cddis.2016.159)
Supplement: Supplementary Informations [file cddis2016159x1.pdf]

PAX7 is a required target for microRNA-206 induced differentiation of fusion-negative rhabdomyosarcoma

Jason A. Hanna, Matthew R. Garcia, Jonathan C. Go, David Finkelstein, Kiran Kodali, Vishwajeeth Pagala, Xusheng Wang, Junmin Peng, and Mark E. Hatley

Supplementary Information Inventory

Supplementary Tables

Supplementary Table S1. Gene ontology functional analysis.
Supplementary Table S2. RMS cell line STR authentication.
Supplementary Table S3. Antibodies used for immunoblot, immunocytochemistry, and immunohistochemistry analysis.
Supplementary Table S4. qRT-PCR primers and probes.
Supplementary Table S5. Primers and cloning sites for 3' UTR cloning of miR-206 and miR-206 targets.
Supplementary Table S6. Mutations of miR-206 target sites in target gene 3' UTR.
Supplementary Table S7. Cloning primers for site directed mutagenesis of miR-206 sites in 3' UTR of target genes.
Supplementary Table S8. Plasmids and PCR primers for subcloning cDNAs.

Supplementary Figure Legends

Supplementary Figures

Supplementary Figure S1. Increased miR-206 levels in RMS induces differentiation and decreases proliferation.
Supplementary Figure S2. miR-206 target identification.
Supplementary Figure S3. miR-206 target validation.
Supplementary Figure S4. Stable cDNA overexpression of targets.
Supplementary Figure S5. Target overexpression and analysis of miR-206 induced differentiation.
Supplementary Figure S6. Reduced exogenous PAX7 expression maintains high proliferation and reduced differentiation.
Supplementary Figure S7. PAX7 overexpression in FN-RMS and FP-RMS.
Supplementary Figure S8. siRNA knockdown of miR-206 targets.
Supplementary Figure S9. Deletion of miR-206 in FN-RMS mouse model.

SUPPLEMENTARY TABLES

Supplementary Table S1. Gene ontology functional analysis.

Group of 23 down-regulated by mRNA, protein and predicted miR-206 targets			
**Category**	**GO term**	**# genes**	**P-value**
GOTERM_BP_FAT	tissue regeneration	3	1.00E-03
GOTERM_BP_FAT	developmental growth	3	7.20E-03
GOTERM_CC_FAT	Golgi apparatus part	4	8.90E-03
GOTERM_CC_FAT	organelle envelope	5	1.20E-02
GOTERM_CC_FAT	cytoskeleton	7	1.20E-02
GOTERM_BP_FAT	regulation of cellular protein metabolic process	4	3.10E-02
GOTERM_BP_FAT	wound healing	3	3.20E-02
GOTERM_CC_FAT	cortical actin cytoskeleton	2	4.10E-02
**Group 39 down-regulated protein and predicted miR-206 targets**			
**Category**	**GO term**	**# genes**	**P-value**
GOTERM_MF_FAT	Ras guanyl-nucleotide exchange factor activity	5	4.70E-05
GOTERM_MF_FAT	lipid binding	7	4.90E-04
GOTERM_MF_FAT	Rho guanyl-nucleotide exchange factor activity	4	6.50E-04
GOTERM_MF_FAT	phospholipid binding	5	6.90E-04
GOTERM_BP_FAT	positive regulation of apoptosis	6	1.20E-03
GOTERM_MF_FAT	nucleoside-triphosphatase regulator activity	6	2.30E-03
GOTERM_CC_FAT	membrane-bounded vesicle	6	3.60E-03
GOTERM_BP_FAT	positive regulation of transcription from RNA polymerase II promoter	5	5.20E-03
GOTERM_MF_FAT	enzyme binding	6	6.40E-03
GOTERM_BP_FAT	RNA biosynthetic process	4	1.90E-02
GOTERM_MF_FAT	phosphoinositide binding	3	2.20E-02
GOTERM_CC_FAT	plasma membrane	13	2.80E-02
GOTERM_BP_FAT	intracellular signaling cascade	7	2.90E-02
GOTERM_MF_FAT	metal ion binding	16	2.90E-02
GOTERM_BP_FAT	positive regulation of nucleobase, nucleoside, nucleotide and nucleic acid metabolic process	5	3.00E-02
GOTERM_BP_FAT	positive regulation of nitrogen compound metabolic process	5	3.30E-02
**Group 33 down-regulated by mRNA and predicted miR-206 targets**			
**Category**	**GO term**	**# genes**	**P-value**
SP_PIR_KEYWORDS	carbohydrate metabolism	3	7.10E-03
GOTERM_CC_FAT	intrinsic to membrane	13	3.70E-02
GOTERM_BP_FAT	phosphate metabolic process	6	4.10E-02
GOTERM_MF_FAT	deaminase activity	2	4.50E-02
**All down-regulated proteins by TMT proteomic profiling**			
**Category**	**GO term**	**# genes**	**P-value**
GOTERM_BP_FAT	cell cycle phase	27	8.90E-08
GOTERM_BP_FAT	organelle fission	17	7.80E-06
GOTERM_CC_FAT	proteinaceous extracellular matrix	20	9.10E-06
GOTERM_CC_FAT	cytoskeleton	49	1.50E-05
GOTERM_BP_FAT	cell division	18	5.00E-05
GOTERM_BP_FAT	regulation of small GTPase mediated signal transduction	16	9.60E-05
GOTERM_BP_FAT	regeneration	8	3.30E-04
GOTERM_BP_FAT	double-strand break repair via homologous recombination	5	3.90E-04
GOTERM_CC_FAT	microtubule organizing center	14	9.60E-04
GOTERM_CC_FAT	centrosome	13	1.00E-03
GOTERM_MF_FAT	cytoskeletal protein binding	21	3.40E-03
GOTERM_BP_FAT	extracellular matrix organization	8	3.70E-03
GOTERM_CC_FAT	cell junction	20	4.00E-03
GOTERM_CC_FAT	anchoring junction	10	5.10E-03
GOTERM_BP_FAT	glutamine metabolic process	4	5.20E-03
GOTERM_BP_FAT	apoptosis	22	5.50E-03

GOTERM_BP_FAT	regulation of cell development	11	5.90E-03
GOTERM_BP_FAT	cell-matrix adhesion	7	6.90E-03
GOTERM_CC_FAT	cell-cell junction	10	9.50E-03
GOTERM_BP_FAT	cytokinesis during cell cycle	3	1.20E-02
GOTERM_CC_FAT	tight junction	6	1.20E-02
GOTERM_BP_FAT	regulation of cell morphogenesis	8	1.30E-02
GOTERM_BP_FAT	cell adhesion	23	1.50E-02
GOTERM_BP_FAT	nucleotide-excision repair	5	2.00E-02
GOTERM_BP_FAT	developmental growth	6	2.50E-02
GOTERM_CC_FAT	actin cytoskeleton	11	3.00E-02
**All down-regulated mRNA by gene array**			
**Category**	**GO term**	**# genes**	**P-value**
GOTERM_BP_FAT	developmental growth	7	1.50E-04
GOTERM_CC_FAT	extracellular matrix	12	2.70E-04
GOTERM_BP_FAT	neural tube development	6	4.10E-04
GOTERM_CC_FAT	basement membrane	6	6.80E-04
GOTERM_BP_FAT	dorsal/ventral pattern formation	5	2.30E-03
GOTERM_BP_FAT	glycoprotein metabolic process	8	2.70E-03
GOTERM_BP_FAT	skeletal system development	10	2.80E-03
GOTERM_BP_FAT	regulation of JUN kinase activity	4	4.80E-03
GOTERM_BP_FAT	neuron fate commitment	4	6.80E-03
GOTERM_BP_FAT	proteoglycan metabolic process	4	7.30E-03
GOTERM_BP_FAT	smoothened signaling pathway	3	1.30E-02
GOTERM_BP_FAT	regulation of cellular response to stress	5	1.50E-02
GOTERM_BP_FAT	extracellular structure organization	6	1.80E-02
GOTERM_CC_FAT	integral to plasma membrane	19	1.90E-02
GOTERM_BP_FAT	embryonic digit morphogenesis	3	2.20E-02
GOTERM_BP_FAT	cell differentiation in spinal cord	3	2.20E-02
GOTERM_CC_FAT	intrinsic to plasma membrane	19	2.30E-02
GOTERM_BP_FAT	tissue morphogenesis	6	2.60E-02
GOTERM_BP_FAT	blood vessel development	7	2.60E-02
GOTERM_CC_FAT	Golgi membrane	6	2.60E-02
GOTERM_BP_FAT	collagen fibril organization	3	2.90E-02
GOTERM_BP_FAT	embryonic organ morphogenesis	5	3.50E-02
GOTERM_BP_FAT	tissue regeneration	3	3.50E-02
GOTERM_BP_FAT	cell fate commitment	5	4.00E-02
GOTERM_BP_FAT	regulation of MAP kinase activity	5	4.20E-02

Supplementary Table S2. RMS cell line STR authentication.[1]

	Cell Line				
	**RD**	**SMS-CTR**	**Rh18**	**Rh30**	**Rh41**
**D3S1358**	15, 17	17	16	15	17
**TH01**	9.3	6	7	9, 9.3	7, 9.3
**D2S11**	28, 29	29, 31.2	32.2, 33.2	29, 31.2	29, 31
**D18S51**	13, 18	13	16, 17	15, 16	15, 16
**Penta E**	12	5, 17	8, 13	7, 17	11, 17
**D5S818**	11	12	12	12, 13	10, 13
**D13S317**	13	11	12	11	8, 9
**D7S820**	8, 12	8, 11	8, 10	10	10, 11
**D16S539**	10, 11	10, 11	9, 12	12	12, 13
**CSF1PO**	10, 11	12	10	10, 11	11, 12
**Penta D**	11, 13	11, 12	7, 12	11, 12	9, 12
**Amelogenin**	X	XY	X	XY	X
**vWA**	18	18, 19	15, 17	17, 18	16, 18
**D8S1179**	11, 15	10, 12	13,15	12, 15	10, 13
**TPOX**	9	8, 12	8, 9	8, 11	8, 11
**FGA**	20,21	21, 22	23	22	20, 22

[1] STR profiles consistent with published profiles
(Hinson A.R. et al., Frontiers in Oncology, 2013, 3:183).

Supplementary Table S3. Antibodies used for immunoblot, immunocytochemistry, and immunohistochemistry analysis.

**Immunoblot antibodies**				
**Antibody**	**Supplier**	**Product No.**	**Dilution**	**Diluent**
CCND2	Cell Signaling Technology	3741	1:1000	5% BSA
EML4	Cell Signaling Technology	12156	1:1000	5% BSA
GAPDH	EMD Millipore	MAB374	1:10000	5% Milk
GJA1	Cell Signaling Technology	3152	1:1000	5% BSA
KIF2A	Sigma	HPA004716	1:1000	5% Milk
NOTCH3	Cell Signaling Technology	5276	1:1000	5% Milk
PAX3	Developmental Studies Hybridoma Bank (DSHB)	Supernatant	1:500	5% Milk
PAX7	DSHB	Supernatant	1:500	5% Milk
PAX7	Sigma	AV32742	1:4000	5% Milk
TWF1	Cell Signaling Technology	8461	1:1000	5% BSA
**Immunocytochemistry antibodies**				
**Antibody**	**Supplier**	**Product No.**	**Dilution**	
MF-20	DSHB	Supernatant	1:25	
Ki67	Thermo Scientific	RM-9106	1:100	
Alexa 488 goat anti-mouse	Invitrogen	A-11029	1:500	
Alexa 568 goat anti-rabbit	Invitrogen	A-11036	1:500	
**Immunohistochemistry antibodies**				
**Antibody**	**Supplier**	**Product No.**	**Dilution**	**Retrieval**
Desmin	ThermoFisher	RB-9014	1:500	HIER
MyoD1	Cell Marque	386R-18	Neat	HIER
Myogenin	Dako	M3559	1:200	HIER
PAX7	DSHB	Supernatant	1:10	HIER

Supplementary Table S4. qRT-PCR primers and probes.

**SYBR Primers**			
**Gene**	**Species**	**Primer 1**	**Primer 2**
ACTA1	Human	CTTCGTCGCACATTGTGTCT	GACAGCGCCAAGTGAAGC
AP1S1	Human	TATGCCAAACCCATCTCCTC	CCAGGACACCTCCAAGAAGA
ATP6V1A	Human	GCACATAACCAAATGTGCTTTC	TCGGTACAGTCTCTGCACCTC
CAP1	Human	CGGTGCATGTCAGAGGTATG	GGAGGTGAGGCGGAACTC
CCND2	Human	ACGGTACTGCTGCAGGCTAT	AGCTGCTGGCTAAGATCACC
CKM	Human	CTCCTTCTCCGTCATGCTCT	GGTGGAGAAGCTCTCTGTGG
EML4	Human	TGACTCAAGAGCTGACAGGC	CGCAAGATGGACGGTTTC
EZH1	Human	ACTATGTGGTGCCTTCTCCG	CATGTGGCACAGACTGCTTC
FAM101B	Human	GCTGGACAGAAACCTCCTCA	GTACCACCATCGTGTACCCC
FAM91A1	Human	CAGGGTCCAAACCAAGAAGG	TCTAGTTGCGCAAACTGCAC
G6PD	Human	CACCAGATGGTGGGGTAGAT	AGAGCTTTTCCAGGGCGAT
GJA1	Human	GAGTTTGCCTAAGGCGCTC	AGGAGTTCAATCACTTGGCG
GJA1(1)	Human	GAGCGCCTTAGGCAAACTC	ACACCTTCCCTCCAGCAGTT
GPD2	Human	GTCCCTTTCACTGCCTTTTG	GAGGAACTGGGTGCTCCTG
HDAC4	Human	CTGGTCTCGGCCAGAAAGT	CGTGGAAATTTTGAGCCATT
IGFBP5	Human	GAGTAGGTCTCCTCGGCCAT	GGTTTGCCTCAACGAAAAGA
KIF2A	Human	ATTGGACGAACATCACCAGC	AGGAATGGCATCCTGTGAAA
LASP1	Human	CATGCGGCTCTTCTCAAACT	AGGTTTCAGCGTAGTGGCAG
MAN1C1	Human	ACCCTCGTAGCCATCTTTTG	AGCGGGAGAAAATCAAGGAG
MYH1	Human	GCACACCCAGAACACCAG	GCTTCTTCCCACCCTTCAG
NOTCH3	Human	CCTGAGTGACAGGGGTCCT	TGTGCAAATGGAGGTCGTT
PAX3	Human	CTTCATCTGATTGGGGTGCT	GACTTGGAGAGGAAGGAGGC
PAX7	Human	CTTCAGTGGGAGGTCAGGTT	CAAACACAGCATCGACGG
PDCD4	Human	TTCATCACCGGAAAAGAGAGA	GTGCAAGCGAAATTAAGGGA
PNP	Human	CAACTTGAGGTCGGTGCTTAG	GTCTGCGAGACCATGGAGA
SLC25A1	Human	ACAATCTCCCTAACCCCGTG	CCCCATGGAGACCATCAA
SULF1	Human	ATCCTGGTTGAATAATCAATCTCT	ATGCAGGTTCTTCAAGGCAG
TAGLN2	Human	CATATGCAGGTCCCCTGTTG	CCCTTGCCTTGAGTCAGTG
TIMP3	Human	ATGGTGTAGACCAGCGTGC	AGGACGCCTTCTGCAACTC
TWF1	Human	ATCCCAGGAATCTGAAGGCT	TCTTTGCCAGAGCCAGAAAT
XPO6	Human	CAGGGCATCTTCGTACCTGT	ATCTGGACGCTGTTTTTGGA
Ccnd2	Mouse	CAGAGCTTCGATTTGCTCCT	ACACACTCACGTGTGATGCC
Ckm	Mouse	CAGCTTGAACTTGTTGTGGG	ACCTCCACAGCACAGACAGA
Myf5	Mouse	GACAGGGCTGTTACATTCAGG	TGAGGGAACAGGTGGAGAAC
Myh2	Mouse	TGCGGAACTTGGATAGATTTG	TTGGTGGATAAACTCCAGGC
Myh4	Mouse	ACTTGGCCAGGTTGACATTG	GCAGGACTTGGTGGACAAAC
MyoD1	Mouse	GTCGTAGCCATTCTGCCG	AGCACTACAGTGGCGACTCA
Myogenin	Mouse	GTGGGAGTTGCATTCACTGG	CTACAGGCCTTGCTCAGCTC
Notch3	Mouse	GAATCTGGAAGACACCCTGG	AAGCGTCTCCTGGATGCTG
Pax3	Mouse	CGGAGCCTTCATCTGACTG	GGCGGATCTAGAAAGGAAGG
Pax7	Mouse	GTCGGGTTCTGATTCCACAT	GCGAGAAGAAAGCCAAACAC
Taqman Probe	**Species**	**Assay ID**	
U6 snRNA	Human/mouse	4427975-001973	
miR-206	Human/mouse	4427975-000510	
18S	Human	4308329	
NOTCH3	Human	Hs01128541_m1	
Myh1	Mouse	Mm01332489_m1	

Supplementary Table S5. Primers and cloning sites for 3' UTR cloning of miR-206 and miR-206 targets.

Gene	3' UTR Region	Fwd Primer	Rev Primer	Sites
**miR-206**		ccccGAATTCggcaaggaggaaagatgcta	ccccGTCGACctggggccagcgaggaggc	
**CCND2**	Stop-1324	gagactcgagggatgccagttgggccgaa	ataagcggccgccacaccctgatgttcagccatc	3
**EGFR**	Stop-1649	gagactcgagccacggaggatagtatgagc	ataagcggccgcgctctgactgatctgggag	1
**EML4**	51-2272	gagactcgagtaaagttcaggtaacaggatgggc	ataagcggccgcgcatgatagcaatggctcaacatc	1
**EZH1**	16-2218	aagctcgagcggcagcacttatggtag	ataagcggccgctaagaggtcacaagccac	0
**G6PD****	55-445			3
**GJA1**	91-1698	gagactcgagtggaggtggtactcaacagc	ataagcggccgcaagaacactctccagaacacatga	2
**HDAC4**	2023-3693	gagactcgagctctgaccacatcgtcaggatag	ataagcggccgcgtcatgccggtcgtacagtc	3
**HEYL**	Stop-2946	gagactcgagatggtccagcagtcgttcc	ataagcggccgcggcacagtcaaccaacaagtaa	2
**IGFBP5**	460-2920	gagagtttaaaccaggatgtggcaatcaatgac	ataagcggccgcttgggaggcttagttccatgt	1
**KIF2A**	Stop-1455	gagactcgagaccggcatttgctgctaaag	ataagcggccgccatagctccccttttggctcc	2
**MAN1C1**	77-977	gagactcgaggattgggaacgaaggccccat	ataagcggccgccactcaactggggtggcca	2
**NOTCH3**	Stop-1014	gagactcgaggacgctcgtcagttcttagatct	ataagcggccgccctttattaggtggtgagggg	1
**PAX3**	Stop-1407	gagactcgaggtgaactgtccacttggagct	ataagcggccgcacttctcttgttccatcaccctc	2
**PAX7**	1499-3097	gagactcgagcctcaattcctcctccctggga	ataagcggccgcgcatagcttggttcttacacatcac	5
**PDCD4**	1932	gagactcgagatataagaactcttgcagtcttaga	ataagcggccgctacagaggatctttacatgtttatt	2
**PIK3C2A**	Stop-3139	gagactcgagactagtgaatgtttgagctttgga	ataagcggccgccaatcgtagtacagttatgaagtc	2
**RAPGEF2**	Stop-1988	gagactcgagggcacagacttttctggaag	ataagcggccgcccagcttgtacaaactggatg	1
**SULF1**	Stop-2203	gagactcgagtcagccccgtctcactgca	ataagcggccgcctgtcatcccaccgatgatacac	2
**TIMP3**	44-1964	gagactcgaggcttcccttggacactaact	ataagcggccgcaatggcatgaccacaatggg	2
**TWF1**	Stop-1812	gagactcgaggtcatcacattaaacattgtaatactag	ataagcggccgccaccagaaggcatgtaattc	2

*miR-206 sites predicted from TargetScan

**G6PD 3'UTR subcloned from Addgene pAG69-G6PD-3'UTR.

Supplementary Table S6. Mutations of miR-206 target sites in target gene 3' UTR.

Gene	Location	Conserved	Original Site*	Mutated Site**
CCND2 Site A	953-959	Yes	AGCAUUGUUAUAAACCAUUCCAU	AGCAUUGUUAUAAACCAUGAAAU
CCND2 Site B	1163-1169	Not mouse	CACCCCUUCCUGCUGACAUUCCC	CACCCCUUCCUGCUGACAUGAAC
CCND2 Site C	1177-1183	Not mouse	GACAUUCCCAUCACAACAUUCCU	GACAUUCCCAUCACAACAUGAAU
EML4 Site A	1439-1445	Yes	UUUACGUGAUUGUGCCAUUCCAA	UUUACGUGAUUGUGCCAUGAAAA
GJA1 Site A	478-485	Yes	UAAGUCCCUGCUAAAACAUUCCA	UAAGUCCCUGCUAAAACAUGAAA
GJA1 Site B	1609-1616	Yes	UUACUAAUUUGUUUGACAUUCCA	UUACUAAUUUGUUUGACAUGAAA
KIF2A Site A	528-534	Yes	CAGCCAAGAAAAAUUACAUUCCU	CAGCCAAGAAAAAUUACAUGAAU
KIF2A Site B	673-679	Yes	AUAUAGCUGCUGGACCAUUCCAU	AUAUAGCUGCUGGACCAUGAAAU
MAN1C1 Site A	211-217	Not mouse	GCCACACUGGCCCACACAUUCCU	GCCACACUGGCCCACACAUGAAU
MAN1C1 Site B	255-261	Not mouse	AGCCCACUCACUUGCCAUUCCAG	AGCCCACUCACUUGCCAUGAAAG
NOTCH3 Site A	658-664	Yes	UCCCUCACUUCACUGCAUUCCAG	UCCCUCACUUCACUGCAUGAAAG
PAX3 Site A	841-848	Yes	CAUCGAGGAGCUAGAACAUUCCA	CAUCGAGGAGCUAGAACAUCATA
PAX3 Site B	1816-1822	Yes	GAGGUAGGCACAAAUACAUUCCU	GAGGUAGGCACAAAUACACCTCU
PAX7 Site A	1588-1595	Not mouse	CUGUCACUGUAAUCUACAUUCCA	CUGUCACUGUAAUCUACAUATGA
PAX7 Site B	1747-1753	Yes	CCCUCAGUCAACAAGACAUUCCU	CCCUCAGUCAACAAGACAUGAAU
PAX7 Site C	2179-2185	Yes	AAACACUCGUCAUCCACAUUCCU	AAACACUCGUCAUCCACAUGAAU
PAX7 Site D	2318-2325	Yes	CAUCCCCAGGAGGCGACAUUCCA	CAUCCCCAGGAGGCGACAUATGA
PAX7 Site E	2984-2990	Not mouse	GUGUUUCAAAUUAUGCAUUCCAG	GUGUUUCAAAUUAUGCAUGAAAG
SULF1 Site A	390-397	Yes	UUGAAUGGAAUAACGACAUUCCA	UUGAAUGGAAUAACGACAUGAAA
SULF1 Site B	871-878	Not mouse	UAAUCACAGCCACCAACAUUCCA	UAAUCACAGCCACCAACAUGAAA
TWF1 Site A	70-76	Yes	CAGGAGAACUGAAAUCAUUCCAU	CAGGAGAACUGAAAUCAUGAAAU
TWF1 Site B	1613-1620	Yes	CAUUAAAGUAAAUGGACAUUCCA	CAUUAAAGUAAAUGGACAUGAAA

*miR-206 recognition site underlined

**nucleotides mutated highlighted in red.

Supplementary Table S7. Cloning primers for site directed mutagenesis of miR-206 sites in 3' UTR of target genes.

Gene	Primer Sequence
EML4 Site A Primer 1	gaatgacagttctgatgcactttttcatggcacaatcacgtaaacgcct
EML4 Site A Primer 2	aggcgtttacgtgattgtgccatgaaaaagtgcatcagaactgtcattc
GJA1 Site A Primer 1	cttaccttcaaagtgcaaattttaacaatttcatgttttagcagggacttaaggacaatc
GJA1 Site A Primer 2	gattgtccttaagtccctgctaaaacatgaaattgttaaaatttgcactttgaaggtaag
GJA1 Site B Primer 1	tgaacatgaccgtagtttaacatttcatgtcaaacaaattagtaattttcattgtacaaaactgtttatg
GJA1 Site B Primer 2	cataaacagttttgtacaatgaaaattactaatttgtttgacatgaaatgttaaactacggtcatgttca
KIF2A Site A Primer 1	ggtaatttacaatgacaattcatgtaatttttcttggctgtgcttcagaaatttagggtc
KIF2A Site A Primer 2	gaccctaaatttctgaagcacagccaagaaaaattacatgaattgtcattgtaaattacc
KIF2A Site B Primer 1	aattccagatttctttacatataagatttcatggtccagcagctatatccactgtc
KIF2A Site B Primer 2	gacagtggatatagctgctggaccatgaaatcttatatgtaaagaaatctggaatt
MAN1C1 Site A Primer 1	cttcatagaaattctctgtagaaattcatgtgtgggccagtgtggccatg
MAN1C1 Site A Primer 2	catggccacactggcccacacatgaatttctacagagaatttctatgaag
MAN1C1 Site B Primer 1	ggtcctttggccctttcatggcaagtgagtgggcttcatagaaa
MAN1C1 Site B Primer 2	tttctatgaagcccactcacttgccatgaaagggccaaaggacc
NOTCH3 Site A Primer 1	aacatgtcccatctttcatgcagtgaagtgagggaggtggggt
NOTCH3 Site A Primer 2	accccacctccctcacttcactgcatgaaagatgggacatgtt
PAX3 Site A Primer 1	atcatcgaggagctagaacatcatatttgcttgtgtgcgtgcgtg
PAX3 Site A Primer 2	cacgcacgcacacaagcaaatatgatgttctagctcctcgatgat
PAX3 Site B Primer 1	tacgtcatagttcttaactctaaaagaggtgtatttgtgcctacctcattcgtgg
PAX3 Site B Primer 2	ccacgaatgaggtaggcacaaatacacctcttttagagttaagaactatgacgta
SULF1 Site A Primer 1	gtgttcagaattcaaatgattaacttctttcatgtcgttattccattcaagtttactctctg
SULF1 Site A Primer 2	cagagagtaaacttgaatggaataacgacatgaaagaagttaatcatttgaattctgaacac
SULF1 Site B Primer 1	gtacccagggtagcttttcatgttggtggctgtgattacctttattagt
SULF1 Site B Primer 2	actaataaaggtaatcacagccaccaacatgaaaagctaccctgggtac
TWF1 Site A Primer 1	caattttttccctactttatatcaacatttcatgatttcagttctcctgtactaaaagctggac
TWF1 Site A Primer 2	gtccagcttttagtacaggagaactgaaatcatgaaatgttgatataaagtagggaaaaaaattg
TWF1 Site B Primer 1	agtttaagactataatcacatctatattctttcatgtccatttactttaatgtagtgtagtggaatttag
TWF1 Site B Primer 2	ctaaattccactacactacattaaagtaaatggacatgaaagaatatagatgtgattatagtcttaaact

Supplementary Table S8. Plasmids and PCR primers for subcloning cDNAs.

**Addgene Plasmids**			
**Gene**	**Catalog Number**	**Fwd Primer**	**Rev Primer**
G6PD	41521	gatccttcgaactagtccatggcagagcaggtgg	ctcaagcttcgaattctcagagcttgtgggggttc
GJA1	27383	gatccttcgaactagtatgggtgactggagcgcct	ctcaagcttcgaattcctagatctccaggtcatcaggcc
IGFBP5	11608	gatccttcgaactagtccaccatggtgttgctcacc	ctcaagcttcgaattctcactcaacgttgctgctgtcg
KIF2A	52401	atatcccgggatggcaacggccaacttcgg	ataagaattcttaaagggcacggggtctctt
NOTCH3-NICD	40640	ataaactagtatggtggcccggcgcaagcgcg	ataagaattctcaggccaacacttgcctcttggg
SULF1	13003	atatcccgggatggcaacggccaacttcgg	atatcccgggttaaccttcccatccatccc
**Open Biosystems Plasmids**			
CCND2	MHS6278-202760158	gagaactagtggagagcgagaccagttttaa	ataagaattcgagaccagattatggacgcg
EML4	MHS6278-213244183	gatccttcgaactagtgagccgggcgacctagag	ctcaagcttcgaattcgcactgaagccagggtgt
HDAC4*	MHS6278-202759515		
PAX3	MHS6278-211689916	gagaactagtagtcgctggaacatttgccc	ataagaattcgcgaagaccagaaacagggc
PAX7	MHS6278-211690930	gagaactagtaagagatcgcagcagggg	ataagaattccatagtacggaagcagaggct
TWF1	MHS6278-202808952	gagaactagtggtcgcgggcggatgacg	atatcccgggttaatcagtagtagcttcagtttccgc

*HDAC4 was blunt cloned using XmaI site.

Supplementary Figure Legends

Supplementary Figure S1. Increased miR-206 levels in RMS induces differentiation and decreases proliferation. (**a**) miR-206 expression in human RMS cell lines and LHCN-M2 immortalized myoblasts and differentiated myotubes. Expression of terminal differentiation genes in human RMS cells 5 days after transfection with NC or miR-206 mimic by qRT-PCR for (**b**) *MYH1*, $p < 0.05$ for all excluding Rh18 $p = 0.20$. (**c**) *ACTA1* $p < 0.05$ for all excluding Rh18 $p = 0.20$, and (**d**) *CKM*, $p < 0.05$ for all excluding Rh18 $p = 0.0664$.

Supplementary Figure S2. miR-206 target identification. (**a**) Heat map comparing differential expression of mRNAs in RD cells transfected with NC and miR-206 mimic. Down-regulated genes indicated in green (-1.5 SD) and up-regulated genes indicated in red (1.5 SD) with FDR < 0.05. (**b**) Volcano plot of the Log_{10} of the p value versus the log ratio fold difference in mRNA expression in RD cells transfected with NC or miR-206 mimic. (**c**) Venn diagram of mRNAs and proteins up-regulated upon miR-206 mimic transfection and predicted targets by the TargetScan algorithm and proteins (**d**) TMT proteomic analysis 24 hours after NC or miR-206 mimic transfection with fold change and (**e**) 30 most down- and 20 most up-regulated proteins with p-value < 0.05 and fold change > 1.3. (**f**) Venn diagram of predicted targets by TargetScan and 24 hour proteomic analysis. (**g**) Gene expression in 77 human FN-RMS tumors compared to 37 normal skeletal muscles of 32 overlapping genes from (f). (**h**) Gene expression in 94 human FP-RMS tumors compared to normal skeletal muscle of 23 overlapping genes from Figure 1e.

Supplementary Figure S3. miR-206 target validation. Luciferase activity in 293T cells co-transfected with wild type 3' UTRs of indicated genes and (**a**) 100 ng of miR-206 or control vector or (**b**) increasing amounts (25, 50 and 100 ng) of miR-206 vector. Student's unpaired t-test $p < 0.0001$ for all excluding EZH1 and empty vector. Luciferase activity represented as mean ± SEM (n = 4), Renilla/Firefly luciferase ratio normalized to empty reporter (no miR-206). (**c**) Schematic of predicted miR-206 target sites. (**d**) qRT-PCR expression for CCND2, (**e**) NOTCH3, (**f**) PAX3, or (**g**) PAX7 in human RMS cells lines 5 days after transfection with NC or miR-206 mimic. Student's unpaired t-test $p < 0.05$ for all comparisons of NC to miR-206 mimic excluding Rh18 cells *CCND2* (p = 0.197), *NOTCH3* (p = 0.0597), and *PAX3* (p = 0.405).

Supplementary Figure S4. Stable cDNA overexpression of targets. (**a**) qRT-PCR validation of cDNA overexpression in RD cells stably transduced with indicated cDNA or control empty control vector 5 days after transfection with NC or miR-206 mimic. (**b**) Immunoblots for cDNA overexpression in RD cells as in (a).

Supplementary Figure S5. Target overexpression and analysis of miR-206 induced differentiation. (**a**) ICC for MHC (green), Ki67 (red), and DAPI (blue) in RD cells stably transduced with indicated cDNA 5 days after transfection with NC or miR-206 mimic. (**b**) Quantification of percentage of MHC expressing cells from (a), $p < 0.01$ for all NC compared to miR-206 mimic transfected and no significant difference between empty vector and cDNA transduced cells with miR-206. (**c**) Quantification of Ki67 positive cells from (a). $p < 0.05$ for CCND2, G6PD, NOTCH3, and PAX3 transfected with miR-206 mimic compared to empty vector with miR-206 mimic. (**d**) qRT-PCR for *CKM* in cells as

described in (a), $p < 0.05$ for CCND2, G6PD, NOTCH3, and PAX3 transfected with miR-206 mimic compared to empty vector cells with miR-206 mimic.

Supplementary Figure S6. Reduced exogenous PAX7 expression maintains high proliferation and reduced differentiation. (**a**) Immunoblot of stably transduced RD cells with empty or increasing viral titer of pSin-PAX7-Blast. (**b**) qRT-PCR and (**c**) immunoblot of PAX7 in stably transduced RD cells with empty or lowest titer of virus (40-fold reduction from Figure 4) 5 days after NC or miR-206 mimic transfection. (**d**) ICC for MHC (green), Ki67 (red), and DAPI (blue) in cells from (c). (**e**) Quantification of percentage of MHC expressing cells from (d), $p < 0.0001$ for miR-206 mimic transfected empty cells compared to PAX7 cells. (**f**) Quantitation of percentage of Ki67 expressing cells from (d), $p < 0.05$ comparing miR-206 mimic transfected empty cells to PAX7 cells. (**g**) Expression of *CKM* by qRT-PCR in cells from (c), $p < 0.05$ comparing miR-206 mimic transfected empty cells to PAX7 cells.

Supplementary Figure S7. PAX7 overexpression in FN-RMS and FP-RMS. (**a**) *PAX7* expression by qRT-PCR in RMS cell lines and normal myoblasts and differentiated myotubes, expression normalized to myoblasts. (**b**) *PAX7* expression by qRT-PCR in SMS-CTR, Rh41, and Rh30 cells transduced with empty or PAX7 cDNA 5 days after transfection with NC or miR-206 mimic. $p < 0.05$ for all Empty NC compared to PAX7 NC, $p < 0.05$ for all Empty NC compared to Empty 206 Mimic, and no significant difference was observed between PAX7 NC and PAX7 206 mimic transduced cells except in SMS-CTR, $p = 0.0118$. (**c**) qRT-PCR expression of *CKM* in cells treated as in (b). For cells transduced with empty control $p < 0.05$ for all Empty NC compared to

Empty 206 mimic. For cells transduced with PAX7, $p < 0.05$ for Rh30 and Rh41 Empty NC compared to PAX7 206 mimic, but $p = 0.482$ for SMS-CTR.

Supplementary Figure S8. **siRNA knockdown of miR-206 targets.** (**a**) Immunoblot and (**b**) qRT-PCR of RD cells 3 days after transfection with indicated siRNAs to CCND2, NOTCH3, PAX3, and PAX7. Student's unpaired t-test $p < 0.05$ for all siRNAs compared to NC. (**c**) ICC for MHC (green), Ki67 (red) and DAPI (blue) of cells transfected with indicated individual siRNAs or a pool of all of the B siRNAs 5 days after transfection. (**d**) Quantitation of ICC for percentage of MHC positive nuclei in cells from (c), $p < 0.05$ for miR-206 mimic, PAX3-B, and PAX7-B compared to NC (**e**) Quantitation for percentage of Ki67 positive nuclei of (c) as well as ICC from Figure 5c, $p < 0.05$ for all excluding CCND2-B ($p = 0.074$), PAX3-B ($p = 0.31$), and PAX7-A ($p = 0.075$) in comparing the siRNAs to the NC siRNA.

Supplementary Figure S9. Deletion of miR-206 in FN-RMS mouse model. (**a**) Schematic of mouse cross breeding to generate *aP2-Cre* tumor mice. (**b**) qRT-PCR in WT and KO tumors for embryonic muscle development genes and terminally differentiated skeletal muscle genes ($n = 3$). No significant differences observed between miR-206 WT and KO tumors. (**c**) Quantification of percentage of MyoD1 positive cells and (**d**) Myogenin positive cells in WT and KO tumors ($n = 2$). Percentage calculated as ratio of positively stained nuclei: total nuclei in 4 random fields from IHC presented in Figure 6D ($p = 0.752$ for MyoD1 and $p = 0.733$ for Myogenin).

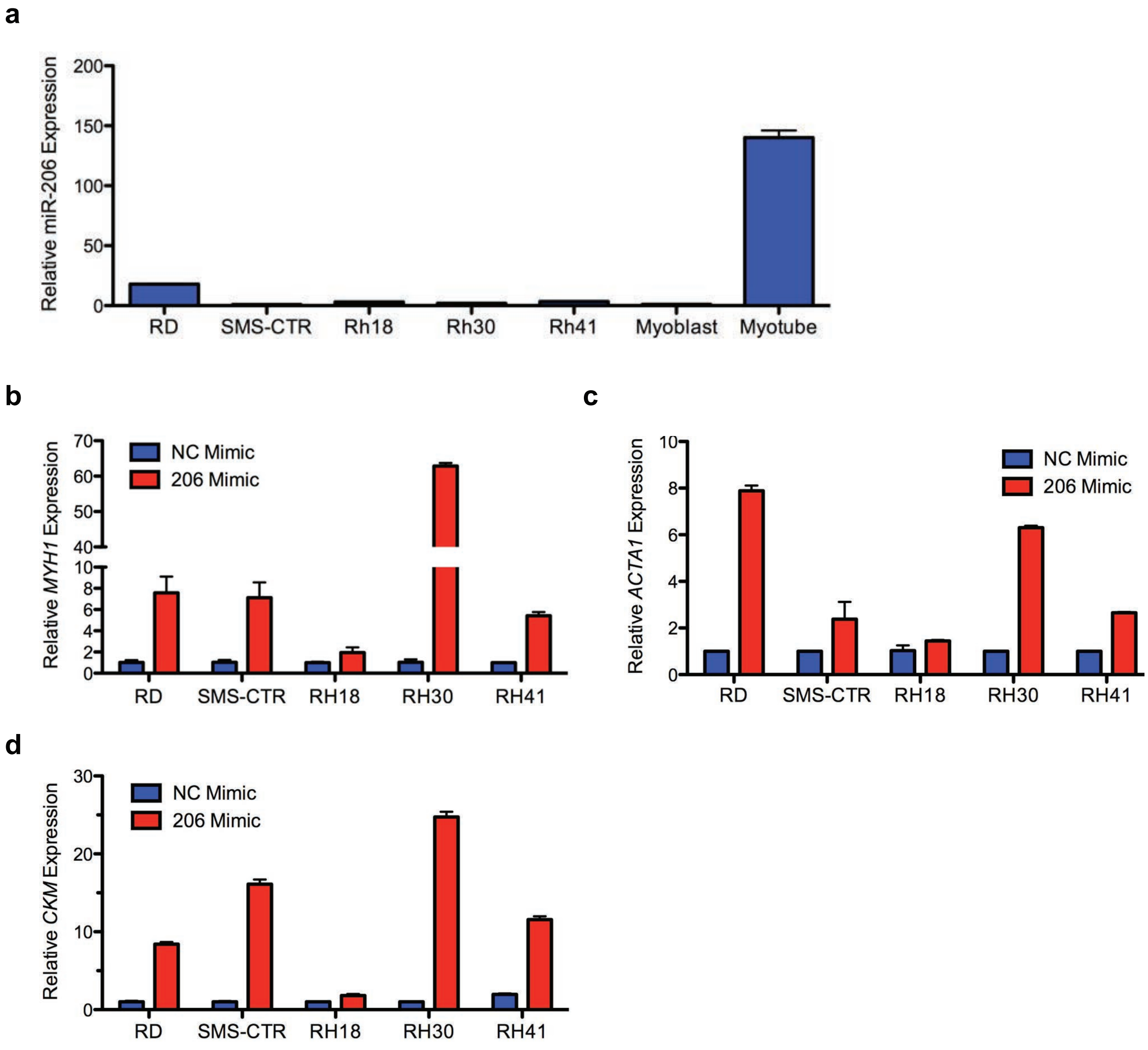

Supplementary Figure S1. Hanna et al.

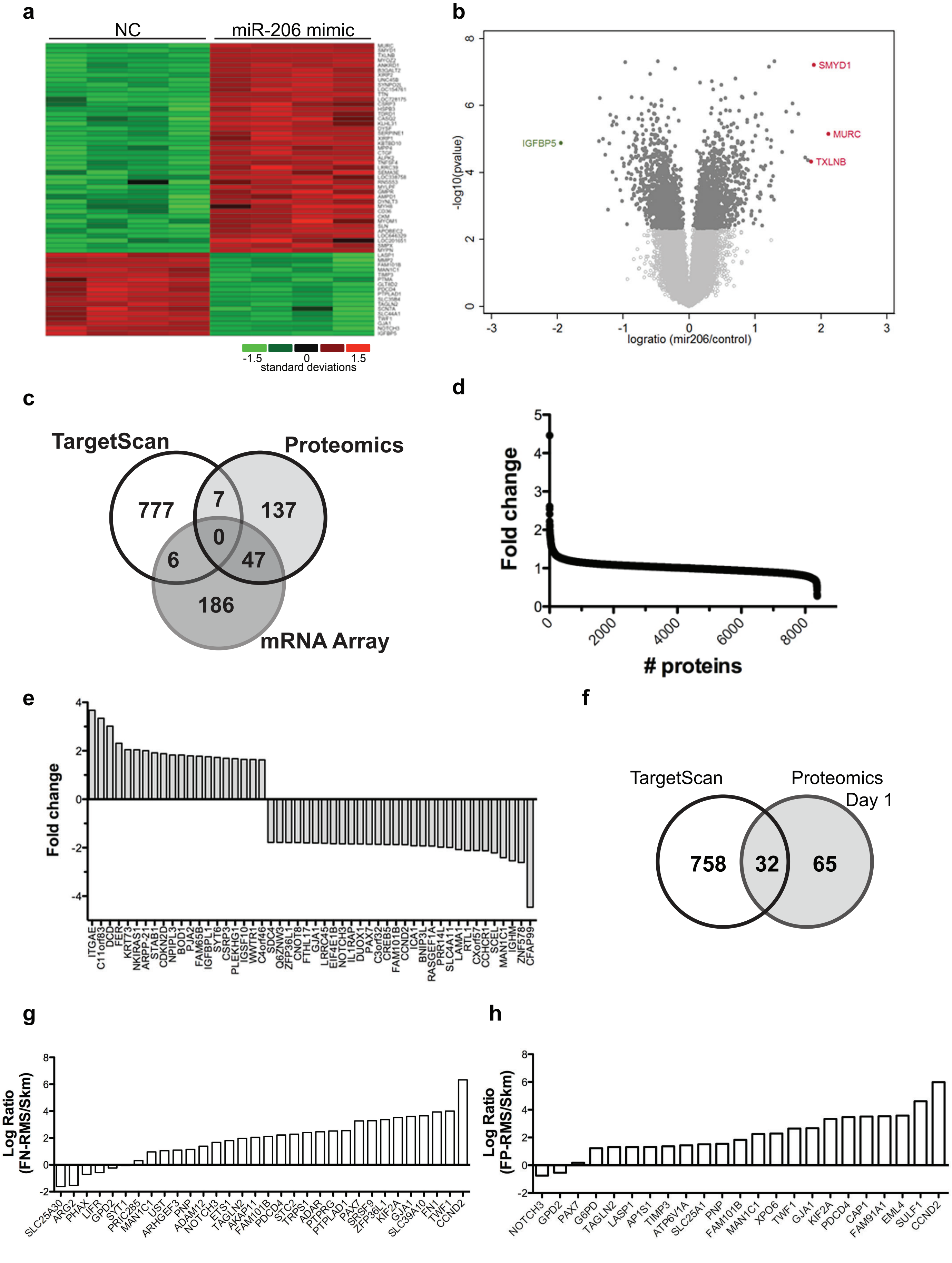

Supplementary Figure S2. Hanna et al.

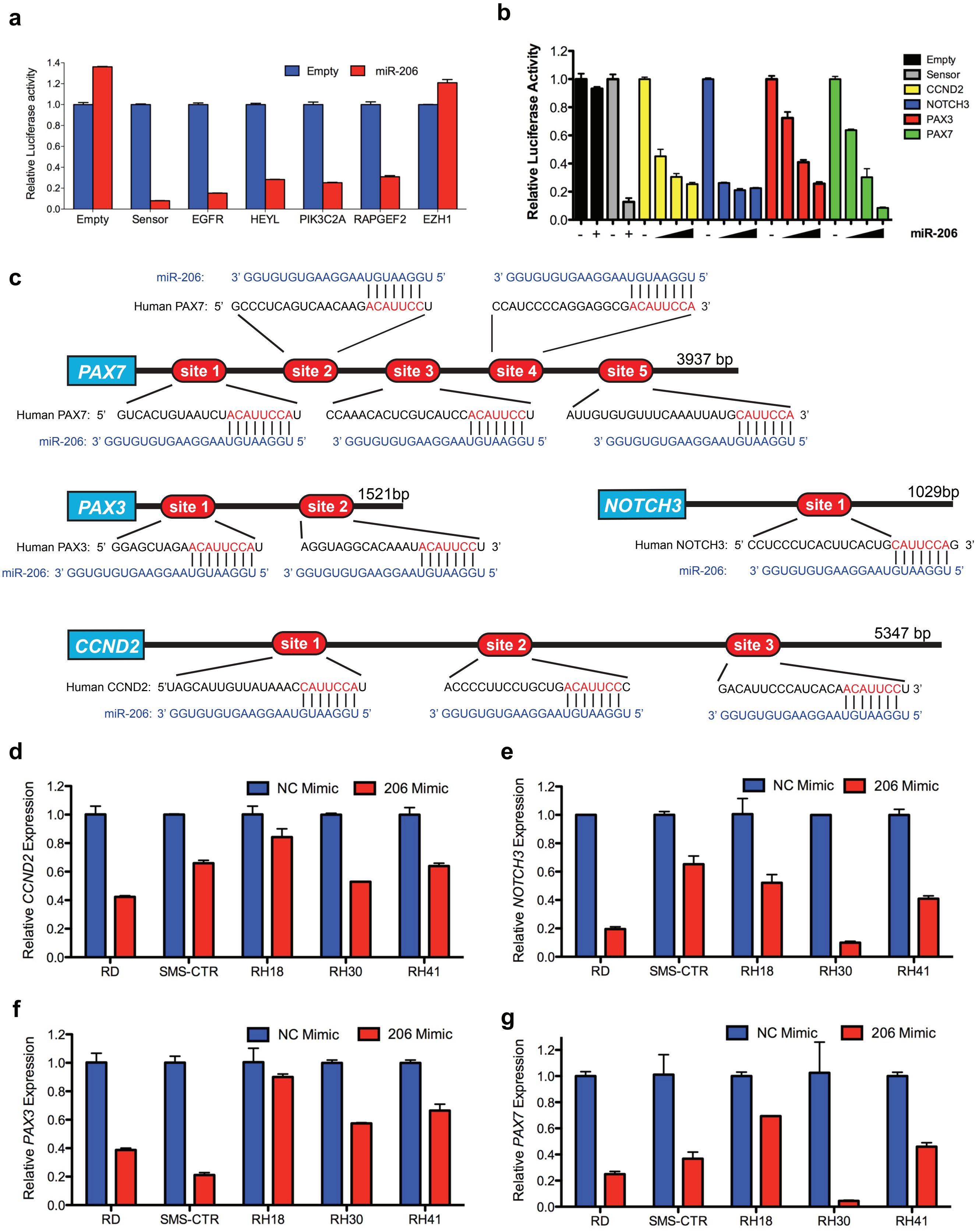

Supplementary Figure S3. Hanna et al.

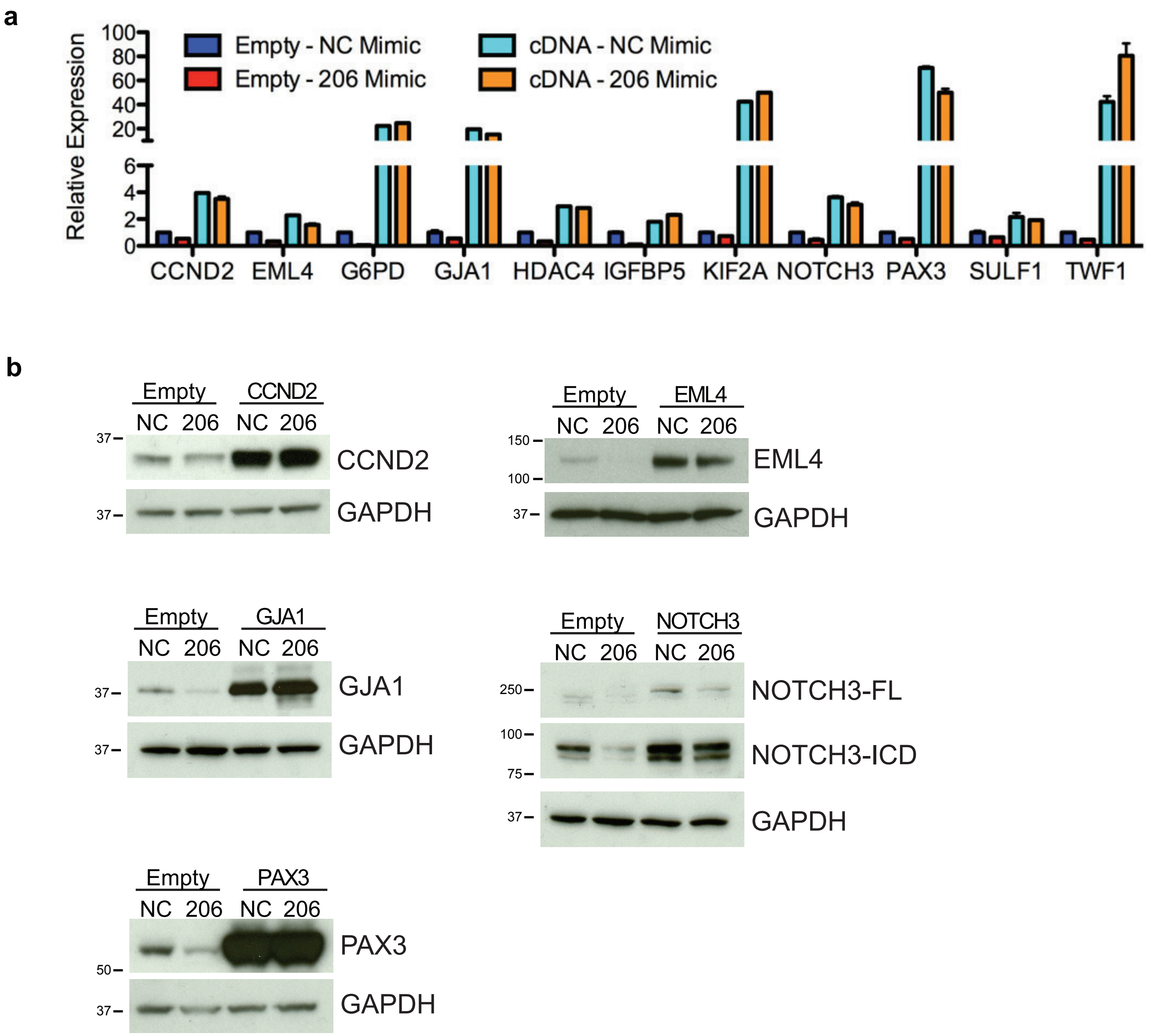
a
Relative Expression
100
80
60
40
20
6
4
2
0
Empty - NC Mimic
Empty - 206 Mimic
cDNA - NC Mimic
cDNA - 206 Mimic
CCND2
EML4
G6PD
GJA1
HDAC4
IGFBP5
KIF2A
NOTCH3
PAX3
SULF1
TWF1
b
Empty
CCND2
NC
206
NC
206
37
CCND2
37
GAPDH
Empty
EML4
NC
206
NC
206
150
100
EML4
37
GAPDH
Empty
GJA1
NC
206
NC
206
37
GJA1
37
GAPDH
Empty
NOTCH3
NC
206
NC
206
250
NOTCH3-FL
100
75
NOTCH3-ICD
37
GAPDH
Empty
PAX3
NC
206
NC
206
50
PAX3
37
GAPDH

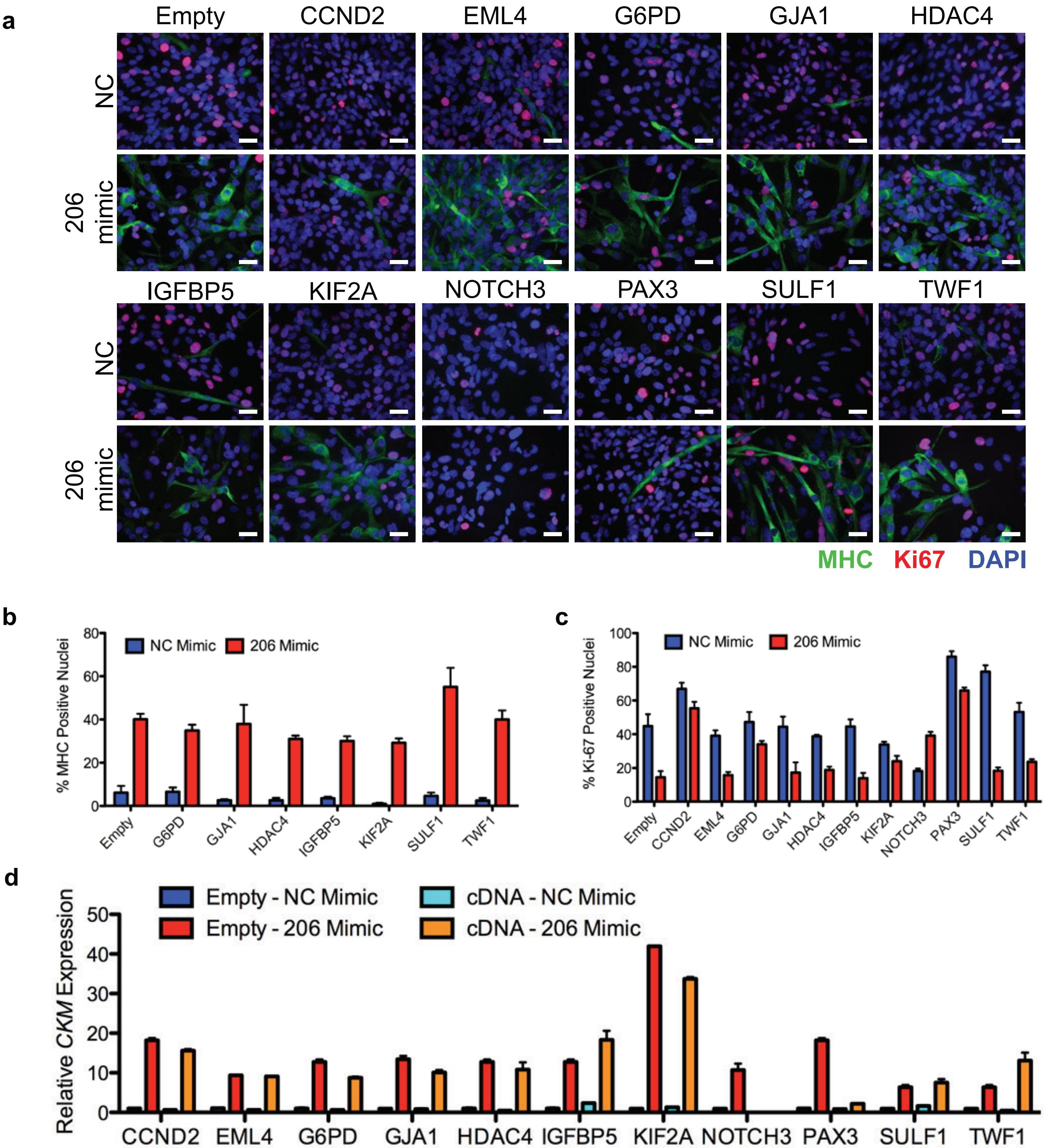

Supplementary Figure S5. Hanna et al.

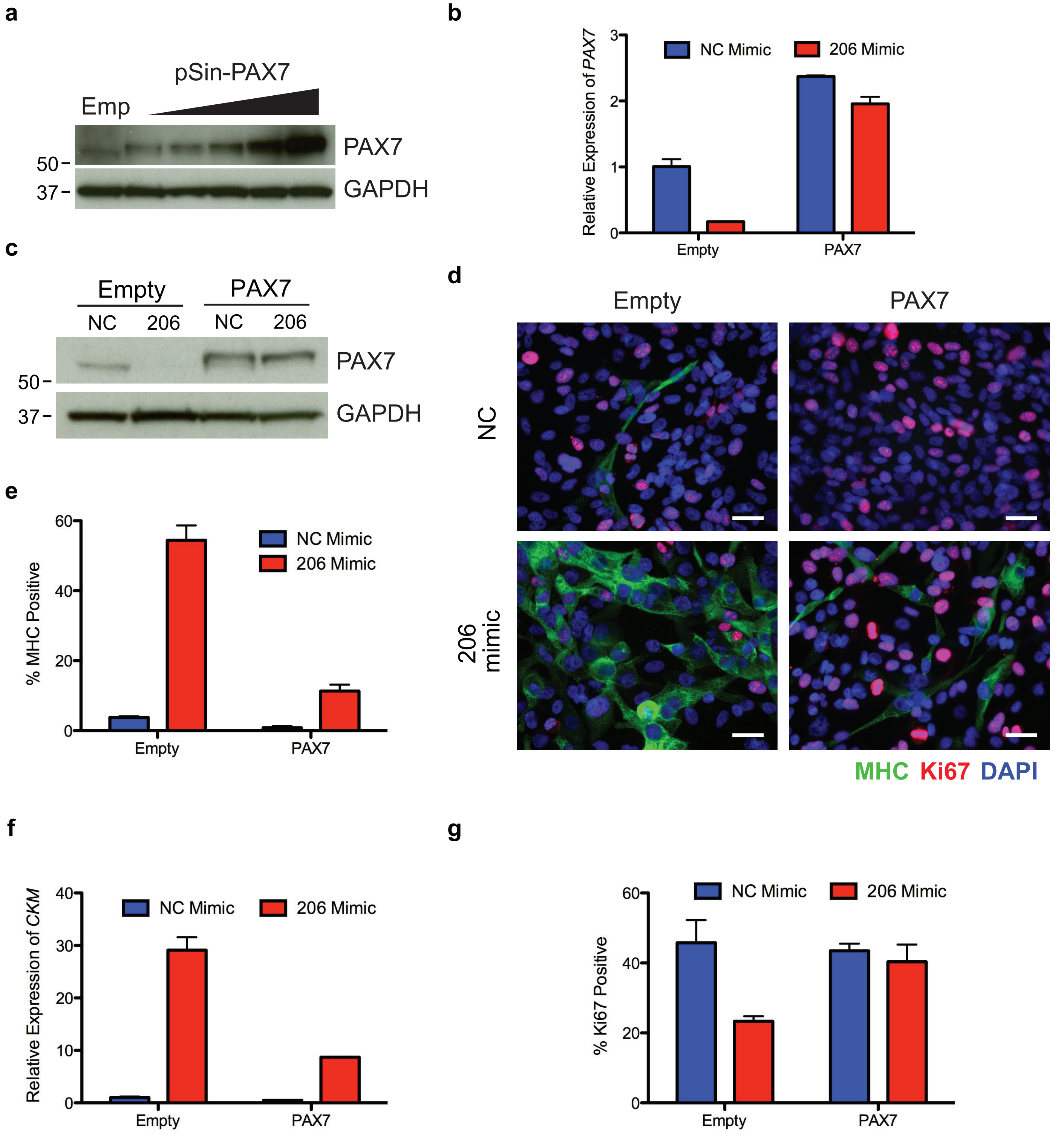

Supplementary Figure S6. Hanna et al.

a

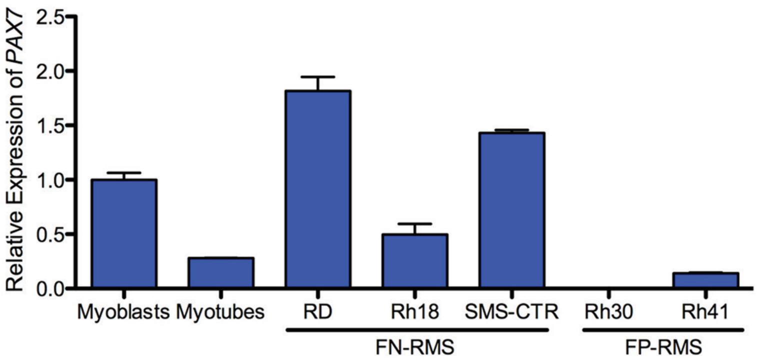

b

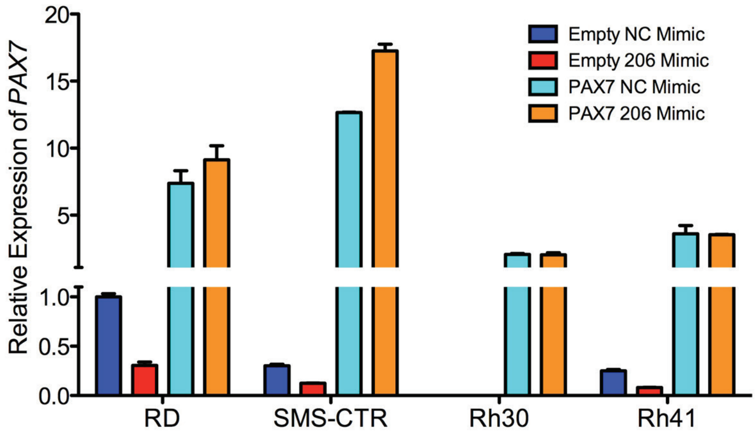

c

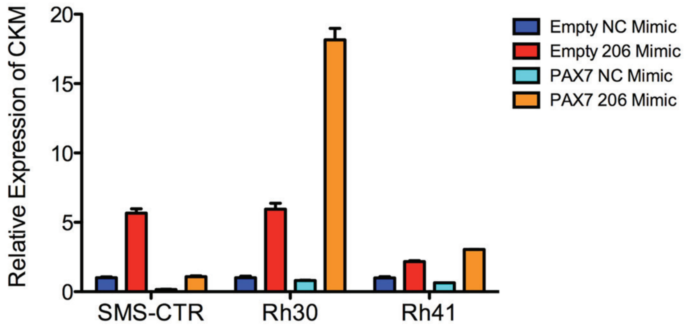

Supplementary Figure S7. Hanna et al.

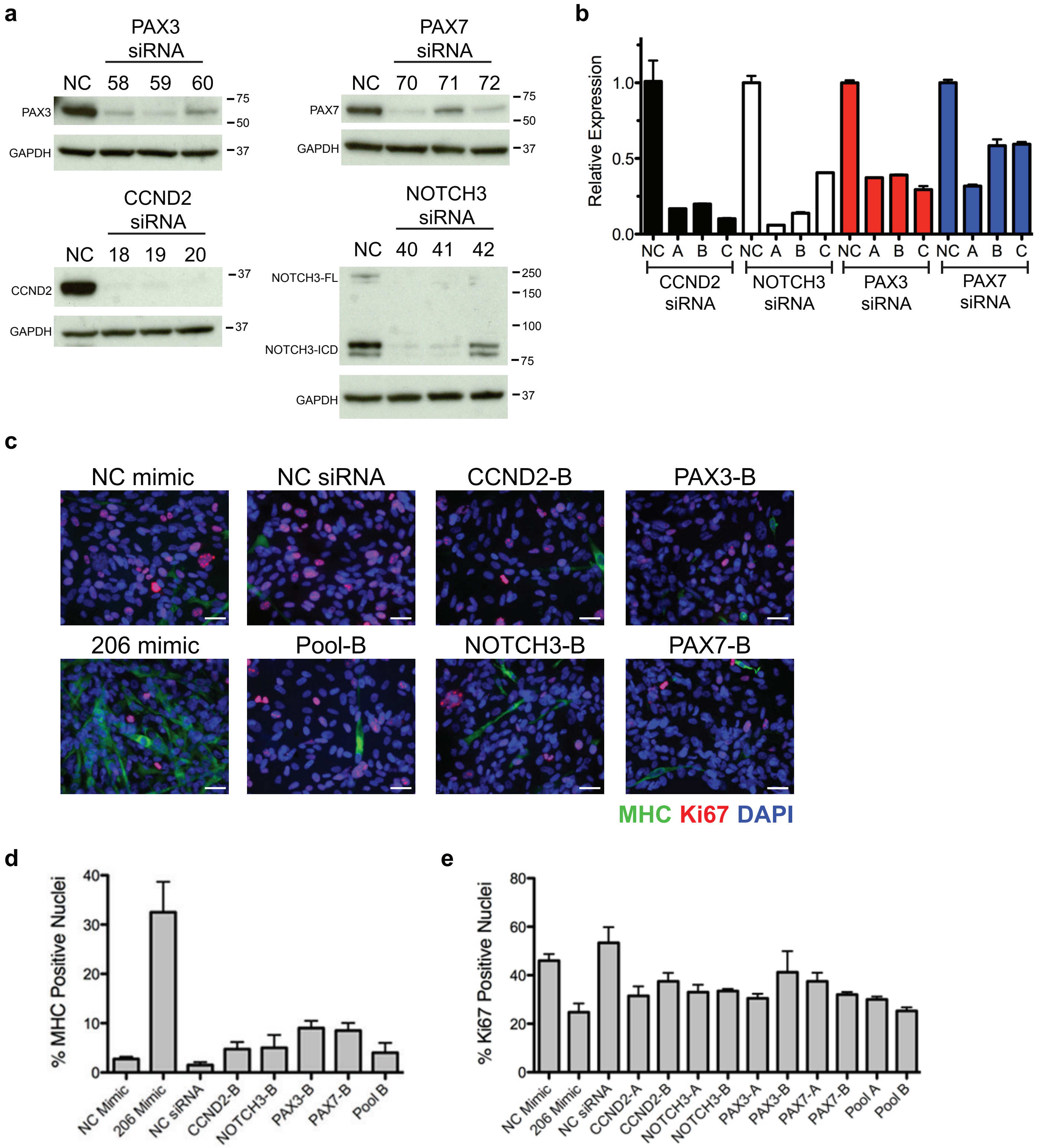

Supplementary Figure S8. Hanna et al.

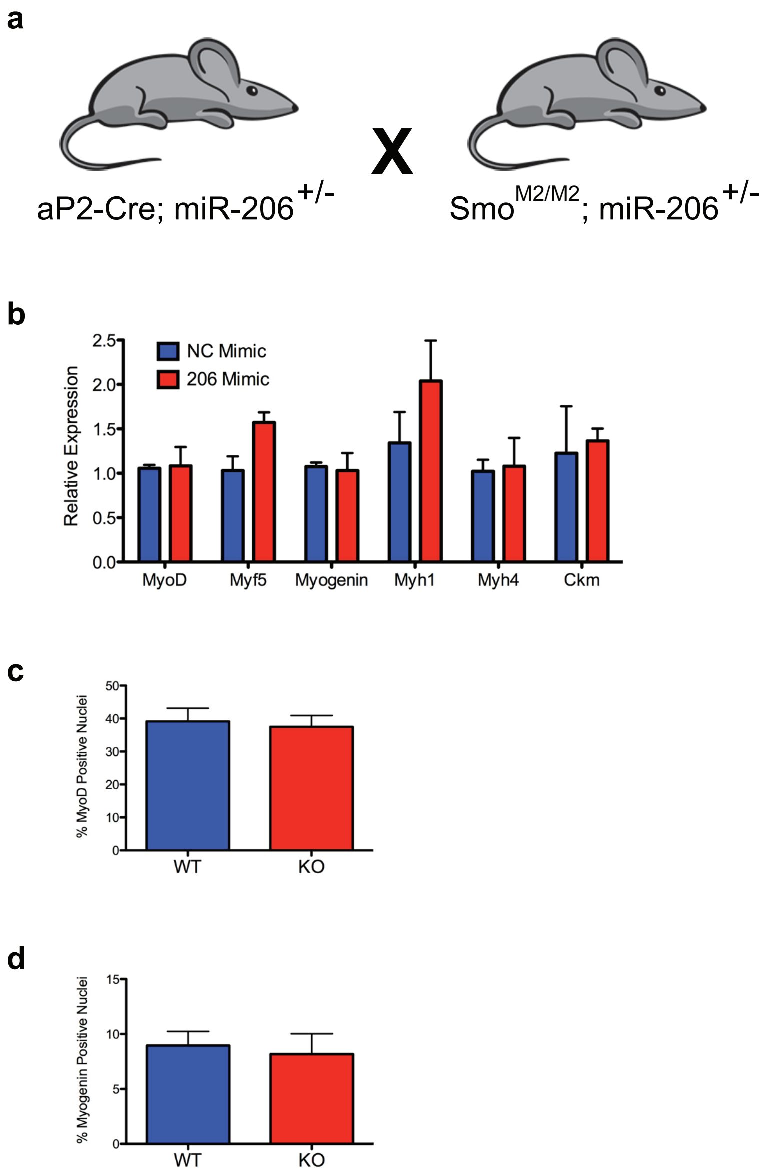

Supplementary Figure S9. Hanna et al.
